# An Approach to the Synthesis of Functionalized Polycyclic Aromatic Hydrocarbons

**DOI:** 10.1002/ejoc.201300750

**Published:** 2013-08-09

**Authors:** Mark Little, He Lan, James Raftery, John J Morrison, Joseph J W McDouall, Stephen G Yeates, Peter Quayle

**Affiliations:** [a] School of Chemistry, University of ManchesterOxford Road, Manchester M13 9PL, UK E-mail: peter.quayle@manchester.ac.uk Homepage: http://www.manchester.ac.uk/research/peter.quayle/

**Keywords:** Benzannulation, Polycycles, Hydrocarbons, Radicals, Cyclization

## Abstract

The application of a new benzannulation reaction for the regiocontrolled synthesis of functionalized chrysenes is reported. The initial benzannulation and the subsequent halogen displacement reactions are both highly regiospecific, which thereby enables the regiocontrolled synthesis of a variety of 4,10-disubstituted chrysenes from commercially available 1,5-dihydroxynaphthalene.

## Introduction

Polyaromatic hydrocarbons (PAHs) have been a focus of research in the field of organic electronics since the first acene semiconductor devices were fabricated from materials such as pentacene and rubrene.^1^ Linear PAHs have since received the majority of attention, with many reporting high-mobility devices.^2^ More recently, numerous reports on the synthesis of linear acenes possessing heterocyclic structures have also appeared, and this area shows much promise for the development of new devices. In contrast, the chemistry of “nonlinear” or “angular” PAHs^3^,4a,4b is much less well developed despite the fact that these PAHs should be more stable than their linear analogues on the basis of Clar's paradigm.^3^,4c,4d Furthermore, few nonlinear PAHs have been investigated as potential semiconductor materials,5a–5c a situation which in part is due to the lack of general synthetic methods in this area.5d,5e

## Results and Discussion

**Scheme 1:**
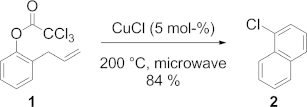
The BHQ reaction.

The synthesis of substituted acenes has focused, until now, on tandem aldol condensations^6^ to afford the corresponding acenequinones, which then may be derivatized and reduced to the acenes, most commonly their ethynyl derivatives.^7^ The development of new synthetic methods leading to the regiospecific synthesis of functionalized, nonlinear polyacenes is imperative if structure–function correlations are to be made for these compounds. In this paper, we present the results of an initial study directed towards the design and synthesis of polyaromatics made readily accessible through our newly developed benzannulation sequence, that is, the Bull–Hutchings–Quayle (BHQ) reaction. The BHQ reaction was discovered by Quayle et al. as a novel means to prepare benzannulated aromatics such as 1-chloronaphthalene (**2**) from 2-allylphenyl trichloroacetate (**1**, Scheme 1).^8^

**Scheme 2:**
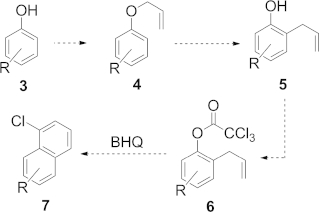
The pathway from a phenol to a polyaromatic aryl chloride.

This new atom-transfer radical cyclization (ATRC)–benzannulation sequence is tolerant of a wide range of common functional groups and exhibits complete regiospecificity for the aryl chloride isomer shown. Moreover, allyl trichloroacetates such as **6** are readily available from phenols by using “textbook” chemistry, and the resulting four-step pathway from phenol **3** to benzannulated product **7** can be readily accomplished on a multigram scale over a short time period (Scheme 2).

**Scheme 3:**
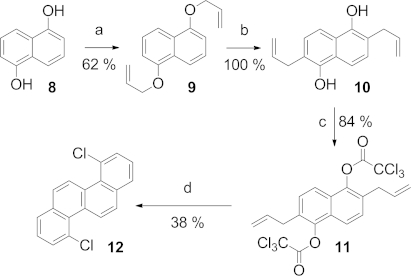
From 1,5-dihydroxynaphthalene to 4,10-dichlorochrysene. Reagents and conditions: (a) allyl bromide (2.4 equiv.), K_2_CO_3_ (2.4 equiv.), acetone, 22 h, r.t.; (b) 210 °C, N_2_, neat; (c) Cl_3_CCOCl, pyridine, Et_2_O, 0 °C, 2 h; (d) CuCl (5 mol-%), diglyme, 162 °C, 2 h.

Many aryl chlorides have been synthesized by using this methodology, including its extension to a “two-directional” double benzannulation sequence. This variant is most appealing if considering the development of new synthetic strategies for PAHs, which would become available from ubiquitous bis(phenols). In this regard, we have shown that 4,10-dichlorochrysene (**12**), a nonlinear phenacene with a novel substitution pattern (Scheme 3), is now readily available from bis(phenol) **8**.

A range of conditions were investigated for the BHQ reaction of **11** leading to **12**; gratifyingly, we found that **12** could be routinely obtained upon thermolysis of **11** in diglyme (b.p. 162 °C) containing CuCl (5 mol-%) in yields ranging from 38 to 43 % (Scheme 3).

**Figure 1:**
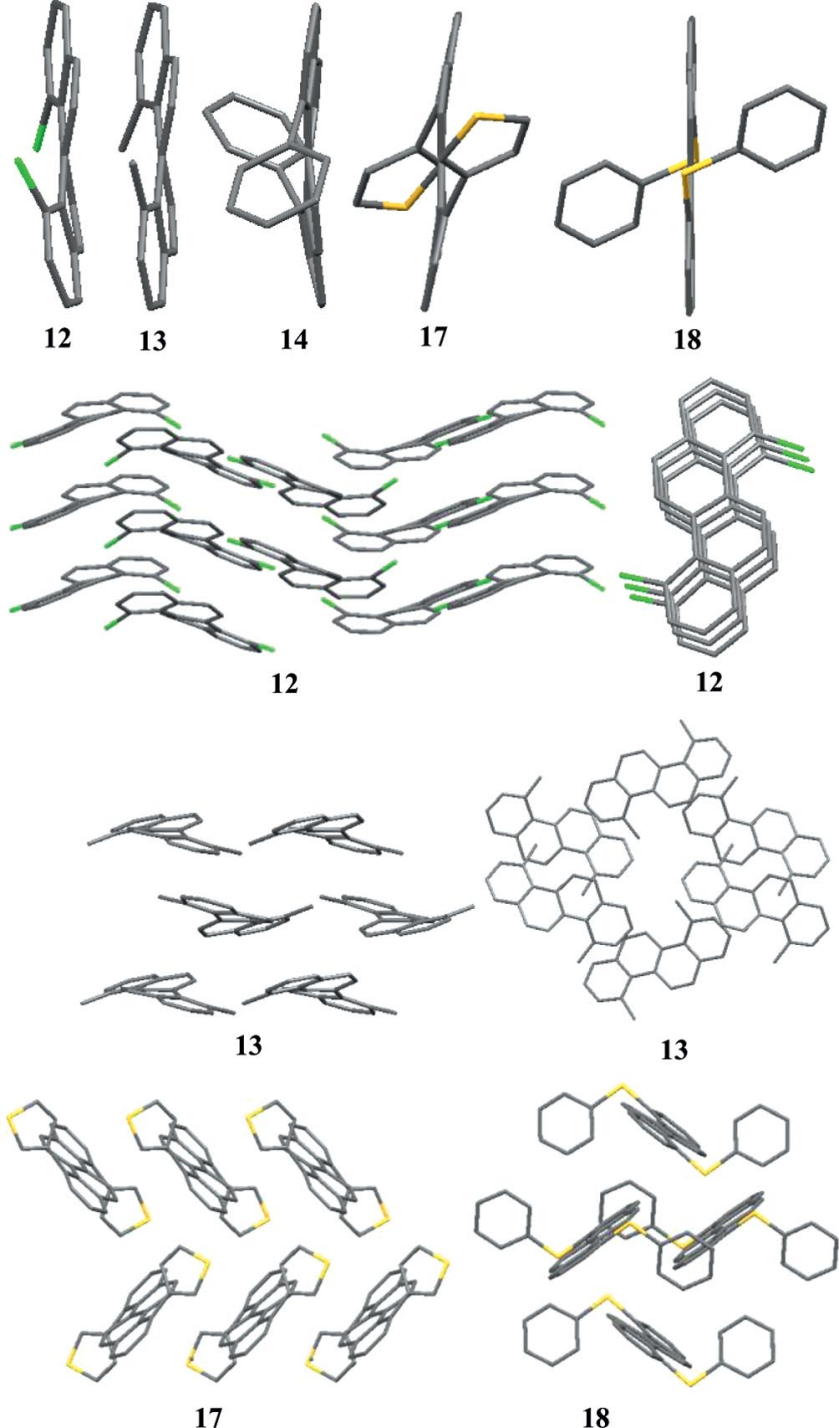
X-ray crystal structures of compounds **12**, **13**, **14**, **17**, and **18** (hydrogen atoms omitted for clarity).

Having optimized the route to **12**, we then wished to investigate its functionalization. Aryl chlorides usually react sluggishly in S_N_Ar or metal-catalyzed displacement reactions,^9^ although we were encouraged by the initial reports of Scott et al.^10^ and more recently by Douglas et al.^11^ concerning the Kumada and Suzuki cross-coupling reactions of related chloroaromatics under the auspices of palladium catalysis.^12^ Significantly, the single-crystal X-ray structure of **12** has a distorted structure (Figure 1), an observation, which apparently was translated into enhanced reactivity of the C–Cl bonds, such that Pd-catalyzed cross-coupling, Cu-promoted coupling, and nucleophilic aromatic substitution reactions are also preparatively viable (Table 1).

**Table 1:** Functionalization of 4,10-dichlorochrysene

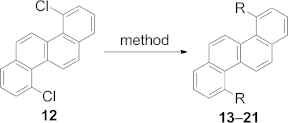			
Compound	R	Method[a]	Yield [%]
**13**	Me	a	78
**14**	Ph	a	91
**15**	*p*-MeOC_6_H_4_	a	69
**16**	1-naphthyl	b	60
**17**	3-thienyl	b	75
**18**	SPh	c	86
**19**	SNp	c	40
**20**	OPh	d	78
**21**	1-octyn-1-yl	e	61
**22**	H	f	–

[a] Method a: RMgBr (4 equiv.), PEPPSI-IPr (5-mol-%), THF (4 mL per 100 mg of **12**), r.t., 30 min. Method b: R(BOH)_2_ (2.2 equiv.), KO*t*Bu (2.2 equiv.), PEPPSI-IPr (2 mol-%), EtOH (1 mL per 100 mg of **12**), r.t., 30 min. Method c: PhSH (2.4 equiv.), K_2_CO_3_ (3 equiv.), DMF (1 mL per 100 mg of **12**), 100 °C, 6 h. Method d: PhOH (2.4 equiv.), Cs_2_CO_3_ (3 equiv.), CuI (2.4 equiv.), diglyme (4 mL per 100 mg of **12**), 150 °C, 48 h. Method e: 1-Octyne (2.2 equiv.), Cs_2_CO_3_ (2.4 equiv.), Pd(PPh_3_)_2_Cl_2_ (6 mol-%), PCy_3_ (15 mol-%), DMF (1 mL per 100 mg of **12**), 110 °C, 40 h. Method f: Isolated as a byproduct of high-temperature coupling. PEPPSI = pyridine-enhanced precatalyst preparation, stabilization, and initiation, IPr = 1,3-bis(2,6-diisopropylphenyl)imidazol-2-ylidene, Cy = cyclohexyl, Np = naphthyl.

Dichloride **12** shows considerable stability to the harsh reaction conditions (>150 °C) of these reactions, and it is resistant towards decomposition even at reaction temperatures in excess of 200 °C for extended periods of time. In spite of this stability, most Pd-catalyzed coupling reactions can be satisfactorily performed without heating, although displacement with oxygen- and sulfur-centered nucleophiles requires somewhat harsher reaction conditions. We were able to prepare a small library of 4,10-disubstituted chrysenes, the structures of which were confirmed by single-crystal X-ray diffraction. Substitution of chrysene in this manner resulted in the molecule adopting a twisted geometry; **12** has a torsional bend of 7.24° about the central bond of the molecule. This bending is not symmetric in **12**, **13**, and **14** but is symmetric in **17** and **18**.^13^ The substitution and the resultant twisting have a profound effect on the crystal packing of the compound; **12** assumes a 1D π–π stacking arrangement with an interlamellar distance of 3.775 Å, which is somewhat longer than the sum of the van der Waals radii. Bis(phenyl) derivative **14** develops a warped 2D brickwork motif dominated by H–π interactions with no observable π–π overlap, whereas both **17** and **18** adopt a familiar herringbone packing, and **18** has a π–π short contact of 3.325 Å. This small selection of chrysene derivatives exemplifies the complex relationship between molecular structure and solid-phase morphology that makes the predictive design of materials so difficult.

**Figure 2:**
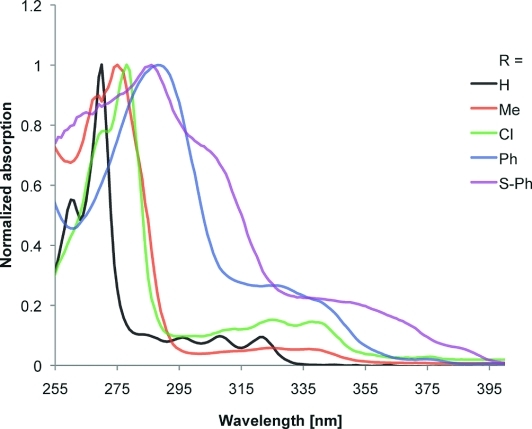
Normalized UV/Vis spectra of selected chrysene derivatives.

To determine the electronic properties of the compounds, they were analyzed by cyclic voltammetry (CV) and UV/Vis spectroscopy. The UV/Vis spectra are characterized by high-intensity absorption between 260 and 300 nm and then a series of lower-intensity bands between 300 and 350 nm (Figure 2). For molecules with more degrees of conformational freedom, these lower-intensity, lower-energy bands are absorbed into the more intense peak.

**Figure 3:**
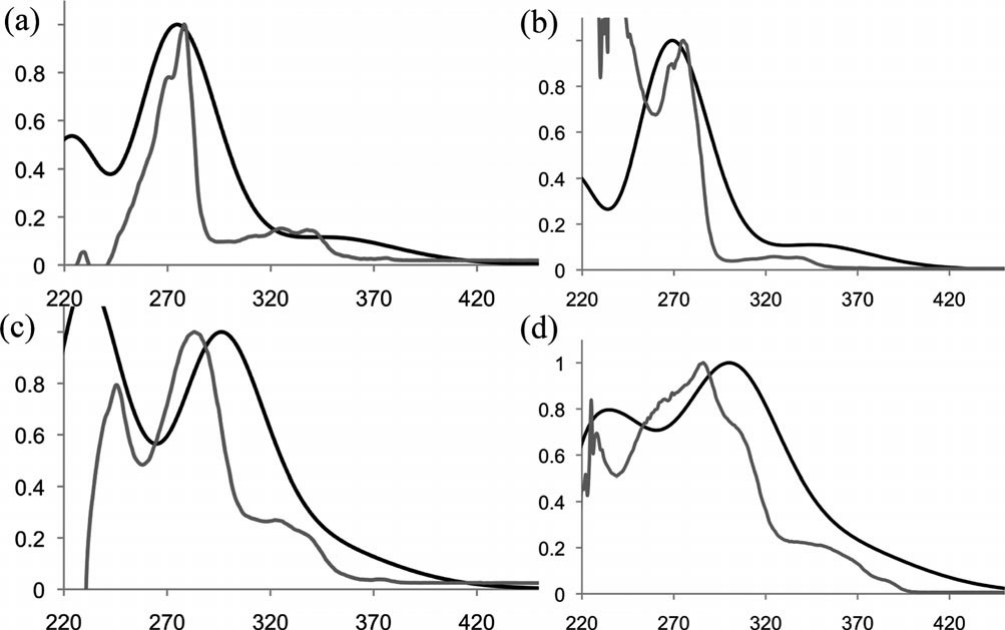
Comparison of the experimentally acquired (gray) and computed (black) UV spectra. (a) **12** (R = Cl), (b) **13** (R = Me), (c) **14** (R = Ph), (d) **18** (R = SPh).

Oxidation potentials were measured by using a ferrocene internal standard, in which Fc/Fc^+^ was taken to be 0.64 V. From this, the energy of the HOMO may be estimated relative to the ferrocene HOMO level of –4.8 eV. The chrysenes described herein display high-energy band-gap transitions and high oxidation potentials, which are typical for nonlinear PAHs. Derivatization of chrysene, as illustrated by this study, has a marked effect upon the electronics of the molecule; values of *λ*_max_ for these compounds range from 270 nm for **13** to 292 nm for **21** (see the Supporting Information for the full data). The oxidation potentials seem to display a relationship with the electronic nature of the substituents, as expected (Table 2). The graphs in Figure 3 show the experimentally acquired UV/Vis absorption curves overlaid with those derived from DFT calculations (Supporting Information). The calculations^14^ suggest that there is no significant delocalization from the chrysene core to the periphery, except in **18** and **19**.

**Table 2:** Oxidation potentials and HOMO energy levels

R	Oxidation potential[a]	HOMO level	
	[V]	Exp.[b]	Calcd.[c]
Cl	1.83	–5.99	–5.92
Me	1.61	–5.77	–5.55
Ph	1.72	–5.88	–5.58
SPh	1.62	–5.78	–5.38

[a] Oxidation potential relative to standard hydrogen electrode (SHE) by using ferrocene as an internal standard for which *V*(Fc/Fc^+^) = 0.64.

[b] Experimental HOMO levels estimated relative to the Fc/Fc^+^ couple for which HOMO(Fc) = –4.8 eV.

[c] B3LYP/6-311G(d,p) calculations based on 6-31G(d,p) geometries.

**Figure 4:**
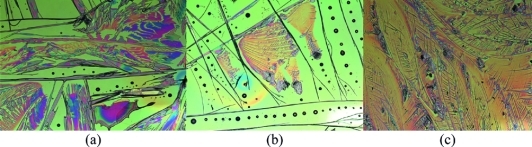
Optical micrograph (200 × 200 μm) of thin films of **12** coated as a 80:20 w/w blend with amorphous statistical copolymers of PS-MeOPS (M_n_ = 6 kDa). (a) Pure PS, (b) 1:1 mol ratio PS/MeOPS, and (c) 1:3 mol ratio PS/MeOPS.

When **12** was spin-coated onto glass from anisole, a noncrystalline film was observed. When spin-coated as a 80:20 w/w blend with amorphous statistical copolymers of styrene (PS) and 4-methoxystyrene (MeOPS; M_n_ = 6 kDa), crystallization of the organic semiconductor was induced (Figure 4).

Although the band gap of **12** is too large to be a useful material for use in a field-effect transistor, it is interesting to note the effect of an amorphous binder on the crystallization of **12**. We ascribe this effect to favorable interactions between slightly electron-deficient **12** and the more electron-rich statistical copolymer of styrene and 4-methoxystyrene. This has previously been reported for linear acenes,^15^ but the effect of fine-tuning the electron density of the binder has previously not been disclosed.

## Conclusions

This preliminary investigation serves as proof of principle and demonstrates that the BHQ benzannulation reaction can be used to generate novel scaffolds, which enables easy access to a range of chrysene derivatives that are not readily accessible by existing methods.^16^ The reactivity of the C–Cl bonds in 4,10-dichlorochrysene enables derivatization of the chrysene core. Application of this basic benzannulation sequence to the synthesis of higher PAHs and investigations into their electronic properties is now in progress. The potential to induce crystallization in thin films of PAHs by judicious blending with amorphous polymeric binders opens up opportunities in controlled crystal engineering and control of the resultant electronic properties.

CCDC-933450 (for **12**), -CCDC-933451 (for **17**), -CCDC-933452 (for **14**), -CCDC-933453 (for **13**), and -CCDC-933454 (for **18**) contain the supplementary crystallographic data for this paper. These data can be obtained free of charge from The Cambridge Crystallographic Data Centre via www.ccdc.cam.ac.uk/data_request/cif.

**Supporting Information** (see footnote on the first page of this article): Full experimental details, including the synthesis, and UV data.

## Supplementary material

As a service to our authors and readers, this journal provides supporting information supplied by the authors. Such materials are peer reviewed and may be re-organized for online delivery, but are not copy-edited or typeset. Technical support issues arising from supporting information (other than missing files) should be addressed to the authors.

## References

[1a] Lin YY, Gundlach DJ, Jackson TN (1996). Device Research Conference, 1996, Digest, 54th Annual.

[1b] Podzorov V, Pudalov VM, Gershenson ME (2003). Appl. Phys. Lett.

[2a] Anthony JE (2008). Angew. Chem.

[b01] (2008). Angew. Chem. Int. Ed..

[2b] Llorente GR, Madec MB, Crouch DJ, Pritchard RG, Ogier S, Yeates SG (2009). Chem. Commun.

[2c] Lin Y, Lia Y, Zhan X (2012). Chem. Soc. Rev.

[2d] Sun Z, Wu J (2012). J. Mater. Chem.

[2e] Zade SS, Bendikov M (2012). J. Phys. Org. Chem.

[2f] Sun Z, Wu J-S (2011). Aust. J. Chem.

[2g] Que H, Chi C (2010). Curr. Org. Chem.

[2h] Zade SS, Bendikov M (2010). Angew. Chem.

[b02] (2010). Angew. Chem. Int. Ed..

[2i] Pho TV, Yuen JD, Kurzman JA, Smith BG, Miao M, Walker WT, Seshadri R, Wudl F (2012). J. Am. Chem. Soc.

[2j] Li G, Wu Y, Gao J, Wang C, Li J, Zhang H, Zhao Y, Zhao Y, Zhang Q (2012). J. Am. Chem. Soc.

[2k] Watanabe M, Chang YJ, Liu S-W, Chao T-H, Goto K, Islam MM, Yuan C-H, Tao Y-T, Shinmyozu T, Chow TJ (2012). Nature Chem.

[3] Wiberg KB (1997). J. Org. Chem.

[4a] Fuchibe K, Jyono H, Fujiwara M, Kudo T, Yokota M, Ichikawa J (2011). Chem. Eur. J.

[4b] Crawford AG, Liu Z, Mkhalid IAI, Thibault M-H, Schwarz N, Alcaraz G, Steffen A, Collings JC, Batsanov AS, Howard JAK, Marder TB (2012). Chem. Eur. J.

[4c] Portella G, Poater J, Bofill JM, Alemany P, Solà M (2005). J. Org. Chem.

[4d] Clar E (1972). The Aromatic Sextet.

[5a] Kunugi Y, Ikari M, Okamoto K (2010). ECS Trans.

[5b] Kawai N, Eguchi R, Goto H, Akaike K, Kaji Y, Kambe T, Fujiwara A, Kubozono Y (2012). J. Phys. Chem. C.

[5c] Wang C, Dong H, Hu W, Liu Y, Zhu D (2012). Chem. Rev.

[5d] Mitchell W, Wang C, D'Lavari M, Blouin N, Tierney S, WO 2012076092 A1 20120614 Appl (2012).

[5e] Pitsinos EN, Vidali VP, Couladouros EA (2011). Eur. J. Org. Chem.

[6] Goodings EP, Mitchard DA, Owen G (1972). J. Chem. Soc. Perkin Trans. 1.

[7] Anthony JE (2006). Chem. Rev.

[8] Bull JA, Hutchings MG, Quayle P (2007). Angew. Chem.

[b03] (2007). Angew. Chem. Int. Ed..

[b04] Bull JA, Hutchings MG, Quayle P (2007). Angew. Chem.

[b05] (2007). Angew. Chem. Int. Ed..

[b06] Bull JA, Hutchings MG, Lujan C, Quayle P (2008). Tetrahedron Lett.

[9] Dhital RN, Kamonsatikul C, Somsook E, Bobuatong K, Ehara M, Karanjit S, Sakurai H (2012). J. Am. Chem. Soc.

[b07] Iglesias MJ, Prieto A, Nicasio MC (2012). Org. Lett.

[10] Quimby JM, Scott LT (2009). Adv. Synth. Catal.

[b08] Scott LT, Jackson EA, Zhang Q, Steinberg BD, Bancu M, Li B (2012). J. Am. Chem. Soc.

[11] Yagodkin, Douglas CJ (2010). Tetrahedron Lett.

[12] Mattarella M, Siegel JS (2012). Org. Biomol. Chem.

[13] Pascal RA (2006). Chem. Rev.

[14] Dreuw A, Head-Gordon M (2005). Chem. Rev.

[b09] Zimmerman PM, Musgrave CB, Head-Gordon M (2013). Acc. Chem. Res.

[15a] Madec MB, Butterworth S, Taboada P, Heenan R, Geoghegan M, Yeates SG (2011). Soft Matter.

[15b] Madec MB, Crouch D, Llorente GR, Whittle TJ, Geoghegan M, Yeates SG (2008). J. Mater. Chem.

[16] Ionkin AS, Marshall WJ, Fish BM, Bryman LM, Wang Y (2008). Chem. Commun.

[b010] Ionkin AS, Marshall WJ, Fish BM, Bryman LM, Wang Y (2009). Chem. Commun.

[b011] Watanabe M, Chen K-Y, Chang YJ, Chow TJ (2013). Acc. Chem. Res.

