# Supplementary material for: An Approach to the Synthesis of Functionalized Polycyclic Aromatic Hydrocarbons
Source: European J Org Chem. 2013 Aug 9;2013(27):6038–41. doi: 10.1002/ejoc.201300750 (PMC4068280; doi:10.1002/ejoc.201300750)
Supplement: Supplementary file 1 [file ejoc2013-6038-SD1.pdf]

Eur. J. Org. Chem. **2013** · © WILEY-VCH Verlag GmbH & Co. KGaA, 69451 Weinheim, 2013 · ISSN 1099–0690

SUPPORTING INFORMATION

DOI: 10.1002/ejoc.201300750
Title: An Approach to the Synthesis of Functionalized Polycyclic Aromatic Hydrocarbons
Author(s): Mark Little, He Lan, James Raftery, John J. Morrison, Joseph J. W. McDouall, Stephen G. Yeates, Peter Quayle*

Contents:

1 - General Remarks

2 - Synthetic Procedures

- 3.0 - 1,5-Bis(allyloxy)naphthalene (9)
- 3.1 - 2,6-Diallyl-1,5-dihydroxynaphthalene (10)
- 3.2 - 2,6-Diallyl-1,5-bis(2,2,2-trichloroacetyl)naphthalene (11)
- 3.3 - 4,10-Dichlorochrysene (12)
- 3.4 - Method A - 4,10-Dimethylchrysene (15)
  - 4,10-Diphenylchrysene (16)
  - 4,10-Bis(4-methoxyphenyl)chrysene (17)
- 4.5 - Method B - 4,10-Bis(1-naphthyl)chrysene (18)
  - 4,10-Bis(3-thienyl)chrysene (19)
- 4.6 - Method C - 4,10-Bis(phenylthio)chrysene (20)
  - 4,10-Bis(2-naphthylthio)chrysene (21)
- 4.8 - Method D - 4,10-Bis(phenyloxy)chrysene (22)
- 4.9 - Method E - 4,10-Bis(oct-1-yn-1-yl)chrysene (23)

3 - UV/vis Spectra

4 - CV Data

- 4.0 - Chrysene (24)
- 4.1 - 4,10-Dichlorochrysene (12)
- 4.2 - 4,10-Dimethylchrysene (15)
- 4.3 - 4,10-Diphenylchrysene (16)
- 4.4 - 4,10-Bis(4-methoxyphenyl)chrysene (17)
- 4.5 - 4,10-Bis(3-thienyl)chrysene (19)
- 4.6 - 4,10-Bis(phenylthio)chrysene (20)
- 4.7 - 4,10-Bis(2-naphthylthio)chrysene (21)
- 4.8 - 4,10-Bis(phenyloxy)chrysene (22)
- 4.9 - 4,10-Bis(Oct-1-yn-1-yl)chrysene (23)

5 - NMR Spectra

- 5.0 - 4,10-Dichlorochrysene (12)
- 5.1 - 4,10-Dimethylchrysene (15)
- 5.2 - 4,10-Diphenylchrysene (16)
- 5.3 - 4,10-Bis(4-methoxyphenyl)chrysene (17)
- 5.4 - 4,10-Bis(1-naphthyl)chrysene (18)
- 5.5 - 4,10-Bis(3-thienyl)chrysene (19)
- 5.6 - 4,10-Bis(phenylthio)chrysene (20)
- 5.7 - 4,10-Bis(2-naphthylthio)chrysene (21)
- 5.8 - 4,10-Bis(phenyloxy)chrysene (22)
- 5.9 - 4,10-Bis(Oct-1-yn-1-yl)chrysene (23)

6 - MO Calculations

1.0 - General remarks.

All reactants and reagents were purchased from Sigma-Aldrich (UK) and were used without further purification. Solvents used were purified by standard methods. All reactions, unless otherwise noted, were carried out under N_2 gas using flame-dried glassware. MWI-assisted reactions were performed with a Biotage Initiator microwave reactor. NMR spectra were acquired using a B400 Bruker Avance III 400 MHz or B500 Bruker Avance II+ 500 MHz spectrometers, using TMS as an internal standard (0.00 ppm). Mass measurements were acquired with a Micromass Trio 200 spectrometer, using electrospray (ES), atmospheric pressure chemical ionisation (APCI) or matrix-assisted laser desorption ionisation (MALDI) techniques, as stated. High resolution mass spectra were recorded on a Kratos Concept IS spectrometer. Ultraviolet-visible (UV-Vis) spectra were recorded on a Varian Cary 50 spectrometer (1 cm cuvette) in de-aerated DCM. Cyclic Voltammetry (CV) was performed on a BASi-Epsilon platform with a scan rate of 100 V/s using solutions of 5 -10 mM of analyte and 100 mM of tetrabutylammonium hexafluorophosphate in DCM. Top-gate bottom-contact organic field effect transistors (OFETs) were fabricated with lithographically defined gold electrodes on glass substrates. The OSC candidate materials were spin-coated as blends with PMS (9 : 1) from ortho-dichlorobenzene at a concentration of 1-7 mg/mL followed by a spin-coated fluoropolymer dielectric material (Lisocon®D139 from Merck Germany).

2 - Synthetic Procedures

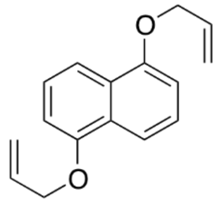

1,5-Bis(allyloxy)naphthalene (9). The allyl naphthyl ether was prepared by adaptation of a standard Williamson ether synthesis procedure. Allyl bromide (32.19 mL, 375 mmol) was added to a stirring suspension of 1,5-dihydroxynaphthalene (25 g, 156.25 mmol)) and K_2CO_3 (51.75 g, 375 mmol) in dry acetone (400 mL) and stirred for 22 h at room temperature. The inorganic components were separated by filtration and the solvent removed in vacuo to afford a brown solid. Diethyl ether (500 mL) was added and the solution extracted with 1M NaOH solution (2 x 150 mL), followed by water (150 mL) and brine (150 mL). The organic phase was dried over $MgSO_4$ and concentrated in vacuo. The crude product was then recrystallized from methanol to afford the product 1,5-bis(allyloxy)naphthalene in 62% yield as a golden solid. m.p. 90 °C. **^{1}H NMR** (400 MHz, CHLOROFORM-d) δ 7.83 (2H, d, J=8.5 Hz, Np-H_4) 7.31 (2H, dd, J=8.5, 7.5 Hz, Np-H_3) 6.78 (2H, d, J=7.5 Hz, Np-H_2) 6.11 (2H, ddt, J=17.5, 10.5, 5 Hz, C-CH=C) 5.45 (2H, ddt, J=17.5, 1.5, 1.5 Hz, C=$CH_{(E)}$) 5.26 (2H, ddt, J=10.5, 1.5, 1.5 Hz, C=$CH_{(Z)}$) 5.0 (4H, dt, J =5, 1.5 Hz, O-CH_2-C) ppm. **^{13}C NMR** (101 MHz, CHLOROFORM-d) δ 154.1, 133.3, 126.8, 125.1, 117.3, 114.5, 105.8, 68.9 ppm. **IR** v_{max} 1590, 1507, 1406, 1379, 1266, 1208, 1109, 1076, 1034, 913, 772, 642 cm^{-1}. **MS** ($APCI^+$) m/z 241 ($[M+H]^+$, 100%), 200 ($[M-C_3H_5]^+$, 40%). **HRMS** (EI^+) $C_{16}H_{16}O_2$ requires 240.1145, found 240.1140.

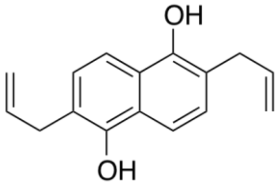

2,6-Diallyl-1,5-dihydroxynaphthalene (10). 1,5-bis(allyloxy)naphthalene was heated neat to 210 °C for 2h under N_2, afording the product in 99% yield. No purification was employed due to the title compound's sensitivity to oxidation. m.p. 135 °C. ^{1}H NMR (500 MHz, CHLOROFORM-d) δ 7.73 (2H, d, J=8.5 Hz, Np-H_4) 7.24 (2H, d, J=8.5 Hz, Np-H_3) 6.09 (2H, ddt, J=18, 10, 6.5 Hz, C-CH=C) 5.51 (2H, s, OH) 5.26 (2H, ddt, J=18, 2, 2 Hz, C=$CH_{(E)}$) 5.25 (2H, ddt, J=10, 2, 2 Hz, C=$CH_{(Z)}$) 3.60 (4H, dt, J=6.5, 2 Hz, Np-CH_2-C) ppm. ^{13}C NMR (101 MHz, CHLOROFORM-d) δ 149.5, 136.2, 127.9, 125.2, 117.9, 117.0, 113.7, 35.7 ppm. IR v_{max} 3310, 1636, 1606, 1496, 1355, 1240, 988, 900, 868, 812, 740, 673 cm^{-1}. MS (EI^+) m/z 240 (M^+, 100%). HRMS (ES^+) $C_{16}H_{17}O_2$ requires 241.1223, found 241.1224.

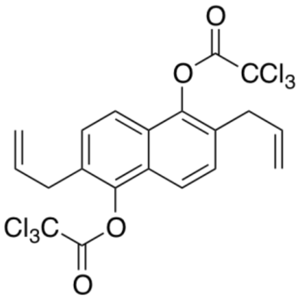

2,6-Diallyl-1,5-bis(2,2,2-trichloroacetyl)naphthalene (11). 2,6-diallyl-1,5-dihydroxynaphthalene (15 g, 62.5 mmol) and pyridine (12.22 mL, 150 mmol) in dry diethyl ether (500 mL) were cooled to 0 °C. To this solution was added trichloroacetyl chloride (16.74 mL, 150 mmol) dropwise with rapid stirring. After 2 h the reaction was quenched by the slow addition of water (200 mL), the organic layer was collected and washed with sat. $NaHCO_3$ soln. (2 x 100 mL), water (2 x 100 mL) and brine (100 mL). The organic fraction was then dried over $MgSO_4$ and concentrated *in vacuo* to afford the title compound in 84% yield. No further purification was employed due to the title compound's instability. m.p. 133 °C. **^{1}H NMR** (500 MHz, CHLOROFORM-*d*) δ 7.74 (2H, d, J=8.5 Hz, Np-H_4), 7.42 (2H, d, J=8.5 Hz, Np-H_3), 5.95 (2H, ddt, J=16.5, 10.5, 6.5 Hz, C-CH=C), 5.16 (2H, ddt, J=10.5, 1.5, 1.5 Hz, C=$CH_{(Z)}$), 5.15 (2H, ddt, J=16.5, 1.5, 1.5 Hz, C=$CH_{(E)}$), 3.52 (4H, dt, J=6.5, 1.5 Hz, Np-CH_2-C) ppm. **^{13}C NMR** (101 MHz, CHLOROFORM-*d*) δ 160.2, 143.6, 134.6, 129.3, 129.2, 126.6, 119.8, 117.5, 89.5, 34.1 ppm. **IR** ν_{max} 1777, 1379, 1191, 1169, 991, 960, 920, 878, 820, 792, 675 cm^{-1}. **MS** (APCI$^+$) m/z 530 ([M($^{35}Cl_5$ + $^{37}Cl_1$)]$^+$, 100%). **HRMS** (EI$^+$) $C_{20}H_{14}O_4Cl_6$ requires 527.9018, found 527.9028.

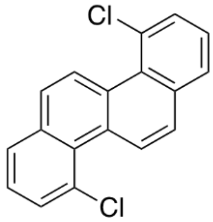

4,10-Dichlorochrysene (12). 2,6-Diallyl-1,5-bis(2,2,2-trichloroacetyl)naphthalene (3 g, 7.58 mmol) and CuCl (5 mol%, 75 mg, 0.76 mmol) were dissolved in diglyme (3 mL) and were thoroughly degassed and purged with N_2. The solution was heated to reflux (162 °C) with gentle stirring for 2 h. The crude product mixture was then directly loaded onto a flash chromatography column and the product eluted with 1:5 DCM/Hexane. The product was isolated as colourless needles in 38% yield. m.p. 159 °C. **^{1}H NMR** (500 MHz, CHLOROFORM-*d*) δ 9.45 (2H, d, J=9 Hz, H_5), 7.83 (2H, dd, J=8, 1.5 Hz, H_1), 7.77 (2H, d, J=9 Hz, H_6), 7.69 (2H, dd, J=8, 1.5 Hz, H_3), 7.46 (2H, t, J=8 Hz, H_2) ppm. **^{13}C NMR** (126 MHz, CHLOROFORM-*d*) δ 134.57, 131.42, 130.21, 129.86, 127.59, 127.47, 126.59, 126.43, 125.00 ppm. **IR** ν_{max} 1416, 1206, 1086, 929, 821, 723, 662 cm^{-1}. **MS** (APCI$^+$) m/z 295 ([M-H]$^+$, 100%,) 296 (M$^+$, 65%). **HRMS** (ES$^+$) $C_{18}H_{10}Cl_2$ requires 296.0148, found 296.0154.

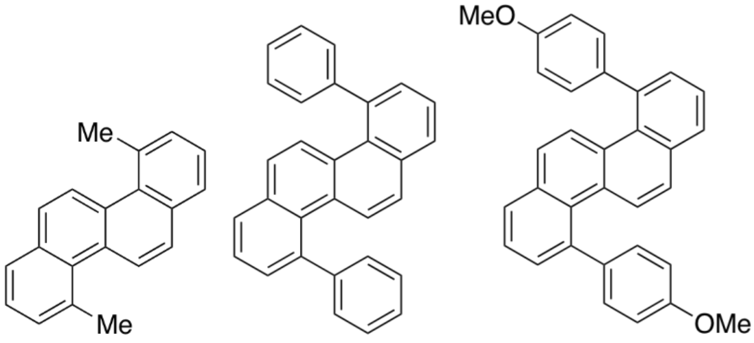

Method A (16, 17, 18). 4,10-Dichlorochrysene (200 mg, 0.68 mmol) and PEPPSI-iPr (5 mol%, 23 mg, 34 μmol) were added to a Schlenk tube equipped with a magnetic stirrer bar and purged with N_2. Thoroughly degassed THF (2 mL) was then added via cannula. With rapid stirring, the corresponding magnesiumbromide in THF (2.72 mmol) was added dropwise, with attention paid to the evolution of a red colour indicative of the activated catalyst species. The crude product was then filtered though silica with DCM to remove traces of palladium.

4,10-Dimethylchrysene (16). Product was isolated as orange crystals in 91% yield after recrystallisation from hexane. m.p. 102 °C. **^{1}H NMR** (500 MHz, CHLOROFORM-*d*) δ 8.59 (2H, d, J=9 Hz, H_5), 7.75 - 7.79 (4H, m), 7.46 (4H, m), 3.05 (6H, s, Me) ppm. **^{13}C NMR** (126 MHz, CHLOROFORM-*d*) δ 135.04, 132.80, 130.97, 130.78, 130.69, 126.46, 126.00, 125.84, 125.42, 26.35 ppm. **MS** (MALDI-Dithranol) m/z 256 (M^+, 19%). **HRMS** (EI^+) $C_{20}H_{16}$ requires 256.1247, found 256.1233.

4,10-Diphenylchrysene (17). Product was isolated as colourless crystals in 84% yield after recrystallisation from DCM/hexane. m.p. 204 °C. **^{1}H NMR** (400 MHz, CHLOROFORM-*d*) *δ* 7.80 (2H, dd, J=8, 1.5 Hz), 7.72 (2H, d, J=9 Hz), 7.59 (2H, t, J=7 Hz), 7.38 - 7.54 (14H, m) ppm. **^{13}C NMR** (101 MHz, CHLOROFORM-*d*) *δ* 145.00, 140.36, 133.03, 130.61, 130.58, 129.19, 128.99, 128.96, 127.73, 127.66, 127.04, 125.80, 124.36 ppm. **MS** (MALDI-Dithranol) m/z 380 (M^+, 95%). **HRMS** (EI^+) $C_{30}H_{20}$ requires 380.1560, found 380.1565.

4,10-Bis(4-methoxyphenyl)chrysene (18). Product was isolated as colourless crystals in 69% yield after recrystallisation from hexane. m.p. 206 °C. **^{1}H NMR** (400 MHz, CHLOROFORM-*d*) *δ* 7.69 - 7.72 (4H, m), 7.40 - 7.53 (4H, m), 7.30 - 7.35 (6H, m), 6.93 (4H, d, J=8.5 Hz), 3.84 (6H, s, O-Me) ppm. **^{13}C NMR** (101 MHz, CHLOROFORM-*d*) *δ* 158.80, 139.98, 137.41, 133.08, 130.74, 130.48, 130.19, 129.10, 127.49, 127.38, 125.77, 124.31, 114.35, 55.36 ppm. **MS** (APCI) m/z 441 ($[M + H]^+$, 100%). **HRMS** (EI^+) $C_{32}H_{24}O_2$ requires 440.1771, found 440.1753.

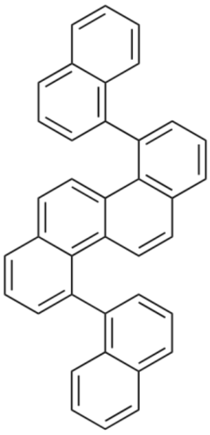 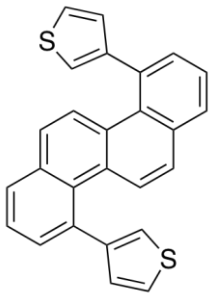

Method B (19, 20). PEPPSI-iPr (2 mol%, 9 mg, 13.6 µmol) and KO^tBu (197 mg, 1.78 mmol) were added to a Schlenk tube equipped with a magnetic stirrer bar and purged with N_2. Thoroughly degassed anhydrous ethanol (3 mL) was added via cannula and the mixture stirred until a colour change from yellow to red was observed, signifying the activation of the catalyst. Without stirring and under a blanket of N_2, 4,10-dichlorochrysene (200 mg, 0.68 mmol) and the corresponding boronic acid (1.62 mmol) were added. The tube was then resealed and stirred under N_2 for 30 min.

4,10-Bis(1-naphthyl)chrysene (19). Product was isolated as a white solid formed as a precipitate which was isolated by vacuum filtration and washing with water (10 mL), methanol (10 mL) and hexane (10 mL). Product is sparingly soluble in chlorinated solvents and toluene. The mono-coupled product is detectable in the ^{1}H NMR as a doublet at 9.55 ppm. 82% yield. m.p. 240 °C. **^{1}H NMR** (400 MHz, CHLOROFORM-d) δ 7.86 - 7.97 (4H, m), 7.70 (2H, dt, J=7.5, 2 Hz), 7.36 - 7.59 (14H, m), 7.07 - 7.24 (4H, m) ppm. **^{13}C NMR** (101 MHz, CHLOROFORM-d) δ 138.15, 138.06, 132.78, 131.54, 131.45, 130.21, 128.30, 128.24, 128.11, 127.63, 126.98, 126.89, 126.35, 126.29, 126.21, 126.10, 126.04, 125.54, 125.28 ppm. **MS** (MALDI-DCTB) 481 ($[M + H]^+$, 100%). **HRMS** (EI^+) $C_{38}H_{24}$ requires 480.1873, found 480.1867.

4,10-Bis(3-thienyl)chrysene (20). Product was isolated as colourless crystals in 60% yield after column chromatography from DCM/hexane. m.p. 195 °C. **^{1}H NMR** (400 MHz, CHLOROFORM-*d*) δ 7.72 - 7.91 (4H, m), 7.55 - 7.69 (4H, m), 7.50 (2H, d, J=9 Hz), 7.38 - 7.44 (4H, m), 7.07 (2H, dd, J=4, 2.5 Hz) ppm. **^{13}C NMR** (101 MHz, CHLOROFORM-*d*) δ 145.3, 134.9, 133.0, 130.6, 130.2, 129.3, 127.9, 126.8, 126.0, 125.8, 124.5, 121.6, 119.8 ppm. **MS** (MALDI-Dithranol) m/z 392 (M^+, 65%). **HRMS** (EI^+) $C_{26}H_{16}S_2$ requires 392.0688, found 392.0671.

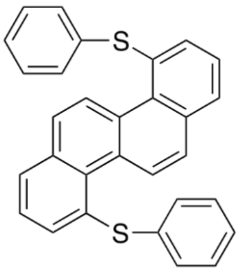 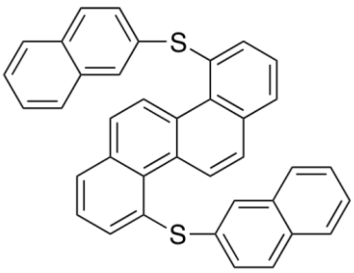

Method C (21, 22). 4,10-Dichlorochrysene (100 mg, 0.34 mmol), potassium carbonate (141 mg, 1.01 mmol), the corresponding thiol (0.81 mmol) and dry DMF (1 mL) were added to a Schlenk tube and purged with N_2. The tube was then heated to 100 °C in a sand-bath for 6 h, until 100% conversion by ^{1}H NMR. The product mixture was then diluted with DCM (10 mL), filtered through a plug of cotton wool and concentrated *in vacuo*.

4,10-Bis(phenylthio)chrysene (21). Product was isolated as a white solid in 86% yield after recrystallization from methanol. The mono-coupled product is detectable in the ^{1}H NMR as a doublet at δ 9.50 ppm. m.p. 178 °C. **^{1}H NMR** (400 MHz, CHLOROFORM-d) δ 9.36 (2H, d, H_5, J=9 Hz), 7.89 (2H, dd, J=8, 1.5 Hz), 7.84 (2H, d, H_6, J=9 Hz), 7.66 (2H, dd, J=7.5, 1.5 Hz), 7.50 (2H, t, H_2, J=7.5 Hz), 7.18 - 7.31 (10H, m, S-Ph) ppm. **^{13}C NMR** (101 MHz, CHLOROFORM-*d*) δ 136.04, 132.58, 132.01, 129.76, 129.27, 128.23, 126.91, 125.97, 125.95, 125.26, 123.95 ppm. **MS** (MALDI-Dithranol) m/z 444 (M^+, 100%). **HRMS** (EI^+) $C_{30}H_{20}S_2$. requires 444.1001 found 444.0994.

4,10-Bis(1-naphthylthio)chrysene (22). Product was isolated as an orange solid in 40% yield after multiple recrystallization from toluene. m.p. 201-203 °C. **^{1}H NMR** (500 MHz, CHLOROFORM-d) δ **^{1}H NMR** (500 MHz, CHLOROFORM-d) d ppm 9.36 (2H, d, J=9 Hz) 7.84 - 7.91 (6H, m) 7.77 - 7.82 (2H, m) 7.69 - 7.74 (4H, m) 7.67 (2H, dd, J=7.5, 1 Hz) 7.44 - 7.49 (6H, m) 7.25 (2H, dd, J=8.5, 1.5 Hz) ppm. **^{13}C NMR** (126 MHz, CHLOROFORM-d) δ ppm 134.47, 133.85, 133.63, 133.50, 133.24, 132.30, 130.93, 130.35, 129.84, 129.04, 128.59, 127.89, 127.75, 127.49, 127.13, 126.61, 126.36, 126.24, 125.10 ppm. **MS** (APCI) m/z 545 ($[M + H]^+$, 100%). **HRMS** (EI^+) $C_{38}H_{24}S_2$. requires 544.1314 found 544.1335.

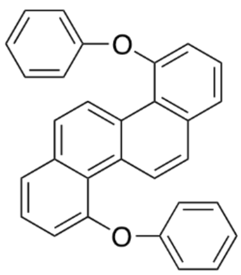

Method D (23). 4,10-Dichlorochrysene (50 mg, 0.17 mmol), cesium carbonate (143 mg, 0.44 mmol), the corresponding phenol (0.40 mmol), copper iodide (75 mg, 0.40 mmol) and diglyme (2 mL) were added to a Schlenk tube equipped with a magnetic stirrer bar and purged under N_2. The tube was then heated to 150 °C in a sand-bath for 48 h, until 100% conversion by ^{1}H NMR. The product mixture was then diluted with DCM (10 mL) and filtered through a plug of silica and concentrated *in vacuo*.

4,10-Bis(phenoxy)chrysene (24). Product was isolated as a white solid in 78% yield after column chromatography with 1:9 EtOAc/hexane. m.p. 216 °C. **^{1}H NMR** (400 MHz, CHLOROFORM-*d*) δ 9.51 (2H, d, H_5, *J*=9.5 Hz), 7.83 (2H, d, H_6, *J*=9.5 Hz), 7.67 (2H, dd, *J*=8, 1.5 Hz), 7.45 (2H, t, H_2, *J*=8 Hz), 7.20 - 7.29 (4H, m, O-Ph), 7.14 (2H, dd, *J*=8, 1.5 Hz), 6.95 - 7.06 (6H, m, O-Ph) ppm. **^{13}C NMR** (101 MHz, CHLOROFORM-*d*) δ 157.61, 155.27, 134.61, 129.89, 129.56, 127.09, 126.65, 126.56, 124.40, 118.56, 118.26, 116.47 ppm. **MS** (APCI) m/z 413 ([M + H]$^+$, 100%). **HRMS** (EI$^+$) $C_{30}H_{20}O_2$. requires 412.1458 found 412.1451.

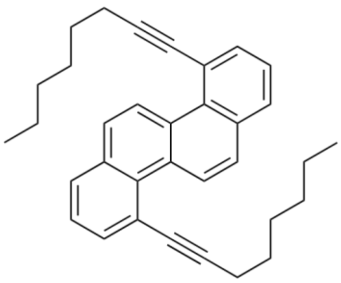

Method E (25). 4,10-Dichlorochrysene (50 mg, 0.17 mmol), the corresponding terminal alkyne (0.36 mmol), cesium carbonate (121 mg, 0.38 mmol), bis(triphenylphosphine)palladium(II) dichloride (6 mol-%, 7 mg, 10 μmol), tri(cyclohexyl)phosphine (15 mol%, 7 mg, 25 μmol) and dry DMF (0.5 mL) were added to a Schlenk tube and purged with N_2. The mixture was then heated to 110 °C in sand-bath for 40 h, until 100% conversion by ^{1}H NMR. The product mixture was then diluted with DCM (10 mL), filtered through a plug of silica and concentrated *in vacuo*.

4,10-Bis(oct-1-yn-1-yl)chrysene (23). The *title compound* was isolated as a waxy orange solid in 61% yield after column chromatography with hexane and recrystallization from hot hexane. 4-oct-1-yn-1-lchrysene is detectable in the ^{1}H NMR spectrum at δ 8.80 and 10.05 ppm as a product of dehalogenation. m.p. 50 °C. **^{1}H NMR** (400 MHz, CHLOROFORM-d) δ 10.22 (2H, d, H_5, J=9 Hz), 7.77 - 7.86 (6H, m, H_1, H_3, H_6), 7.46 (2H, t, H_2, J=7.5 Hz), 2.56 (4H, t, C≡C-CH_2, J=7 Hz), 1.70 (4H, quin, J=7.5 Hz), 1.52 (4H, quin, J=7.5 Hz), 1.25 - 1.38 (4H, m), 1.19 (4H, m), 0.76 - 0.92 (6H, m) ppm. **^{13}C NMR (**125 MHz, CHLOROFORM-d) δ 134.73, 132.96, 130.15, 129.81, 128.43, 125.82, 125.49, 125.20, 120.57, 96.43, 83.40, 31.54, 28.90, 28.62, 22.67, 20.16, 14.15 ppm. **MS** (APCI) m/z 445 ([M + H]$^+$, 100%), 477 ([M + Na]$^+$, 85%). **HRMS** (EI$^+$) $C_{34}H_{36}$. requires 444.2812 found 444.2826.

A major by-product of this reaction was (*E*)-hexadec-7-en-9-yne: **^{1}H NMR** (400 MHz, CHLOROFORM-d) δ 5.95 (1H, dt, J=16, 6 Hz), 5.35 (1H, d, J=16 Hz), 2.18 (2H, m), 1.99 (2H, m), 1.10 - 1.50 (16H, m), 0.84 (6H, m).

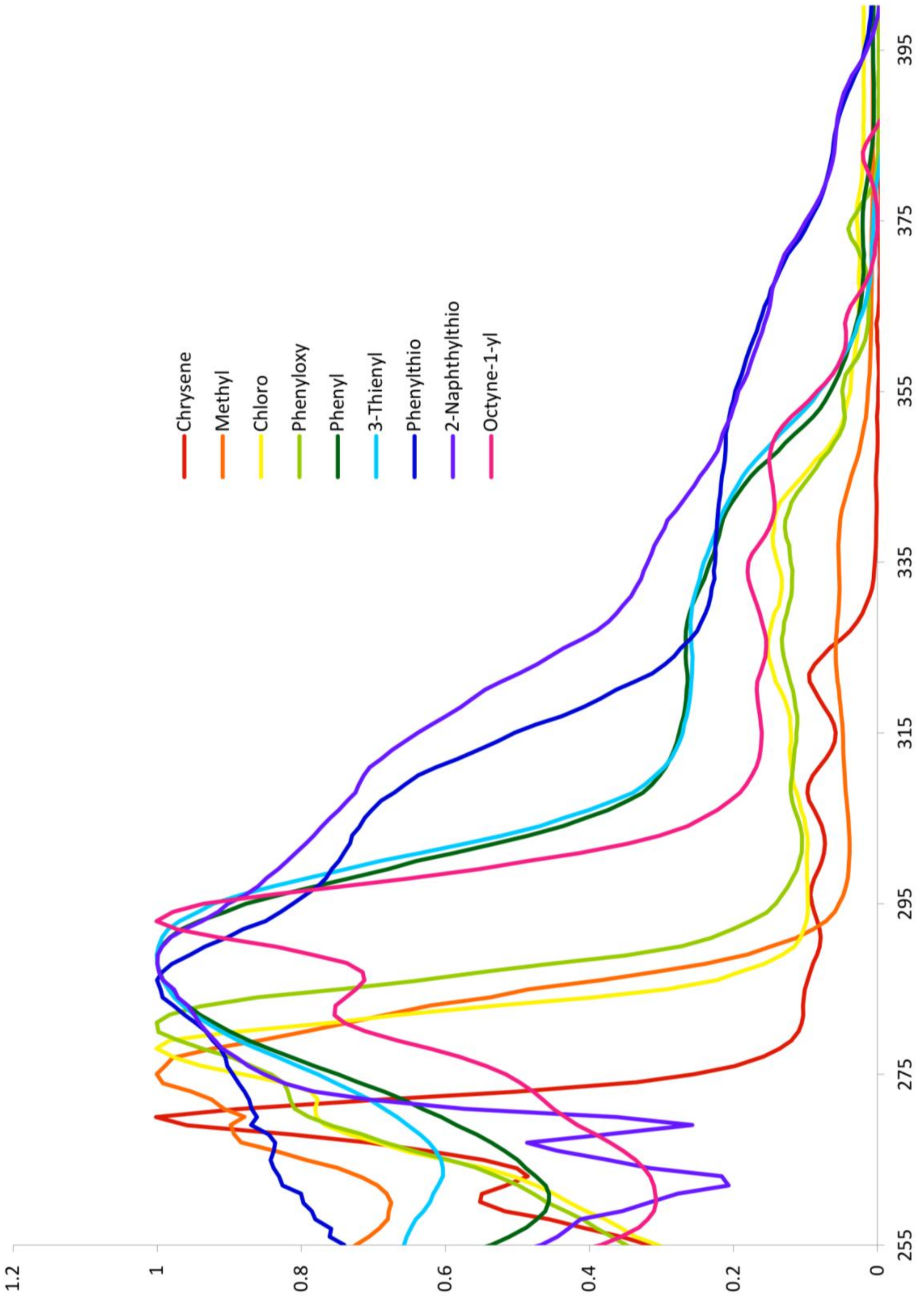

Chrysene
Methyl
Chloro
Phenyloxy
Phenyl
3-Thienyl
Phenylthio
2-Naphthylthio
Octyne-1-yl
255
275
295
315
335
355
375
395
0
0.2
0.4
0.6
0.8
1
1.2

Section 4 - Cyclic voltammetry of 4,10-chrysene derivatives

4.0 - Chrysene (24)

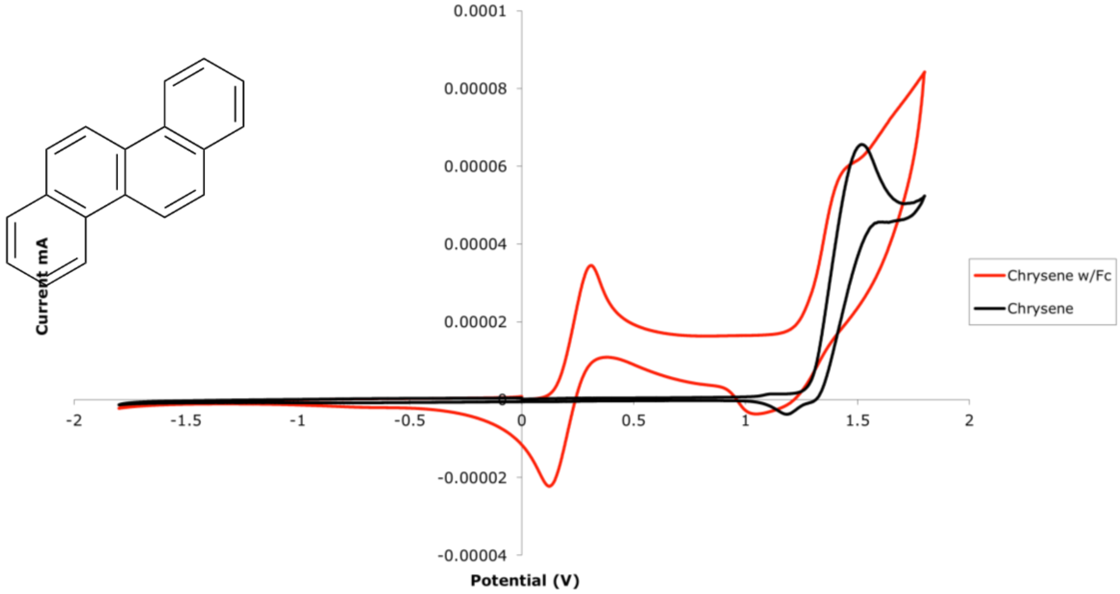

4.1 - 4,10-Dichlorochrysene (12)

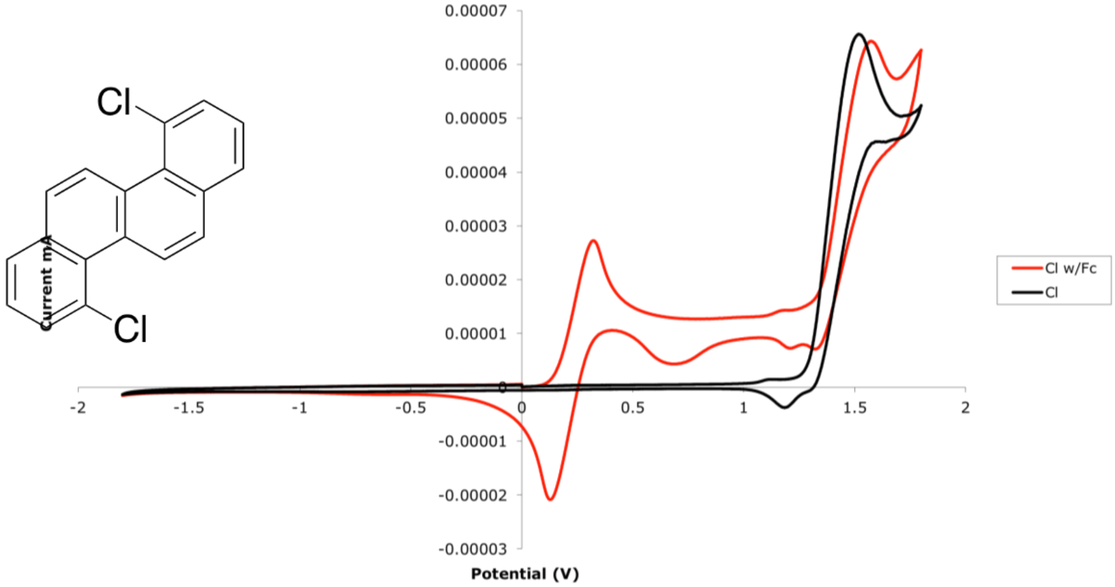

4.2 - 4,10-Dimethylchrysene (15)

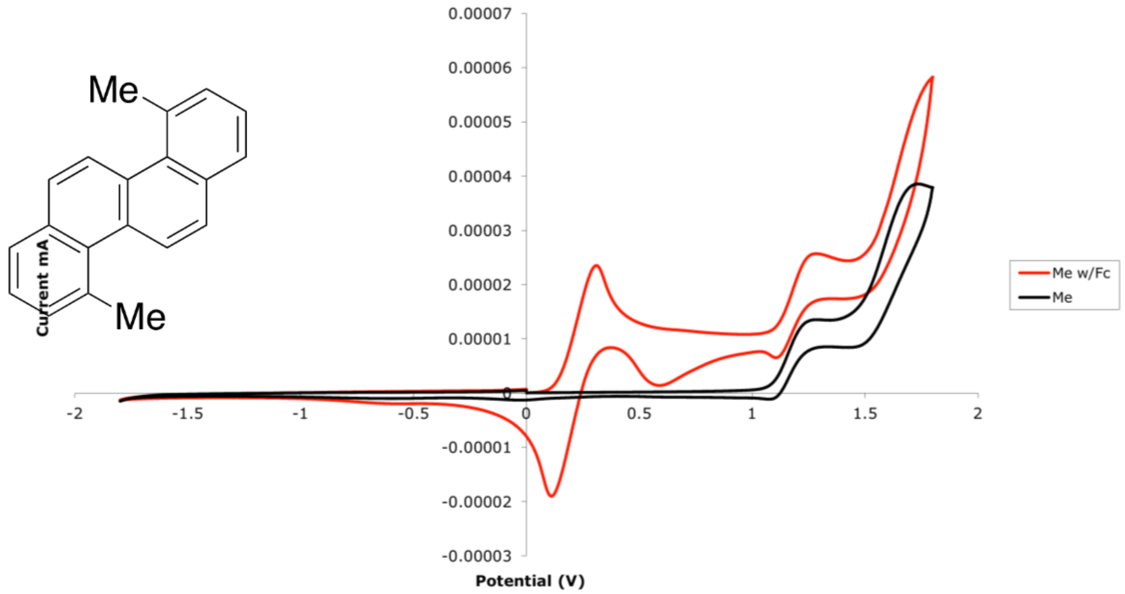

4.3 - 4,10-Diphenylchrysene (16)

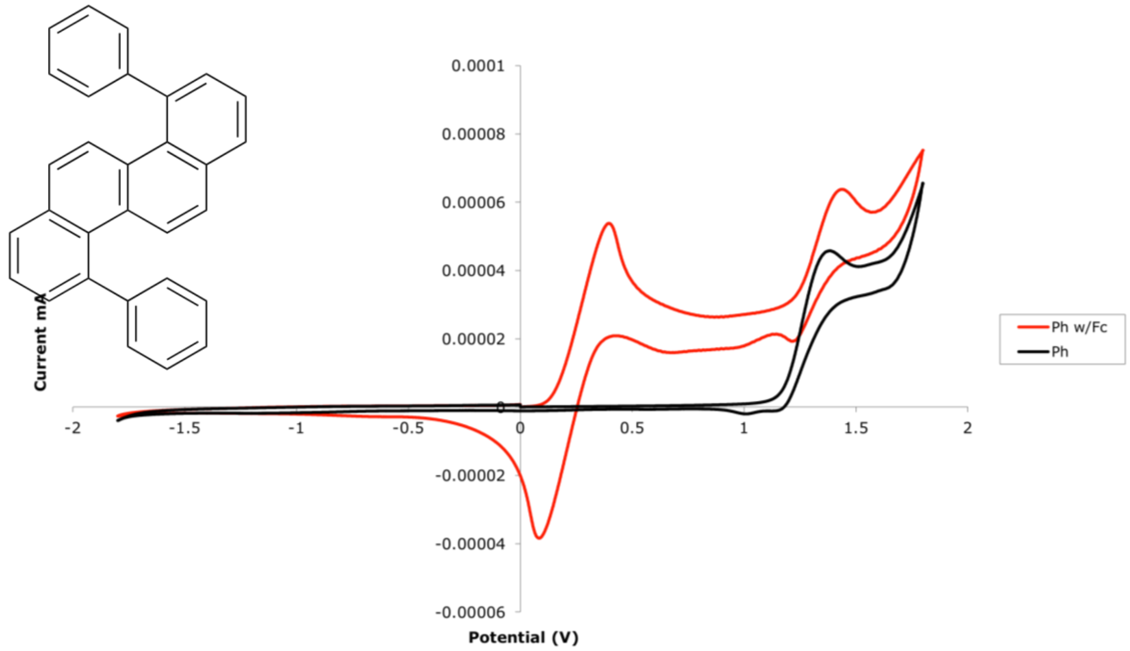

4.4 - 4,10-Bis(4-methoxyphenyl)chrysene (17)

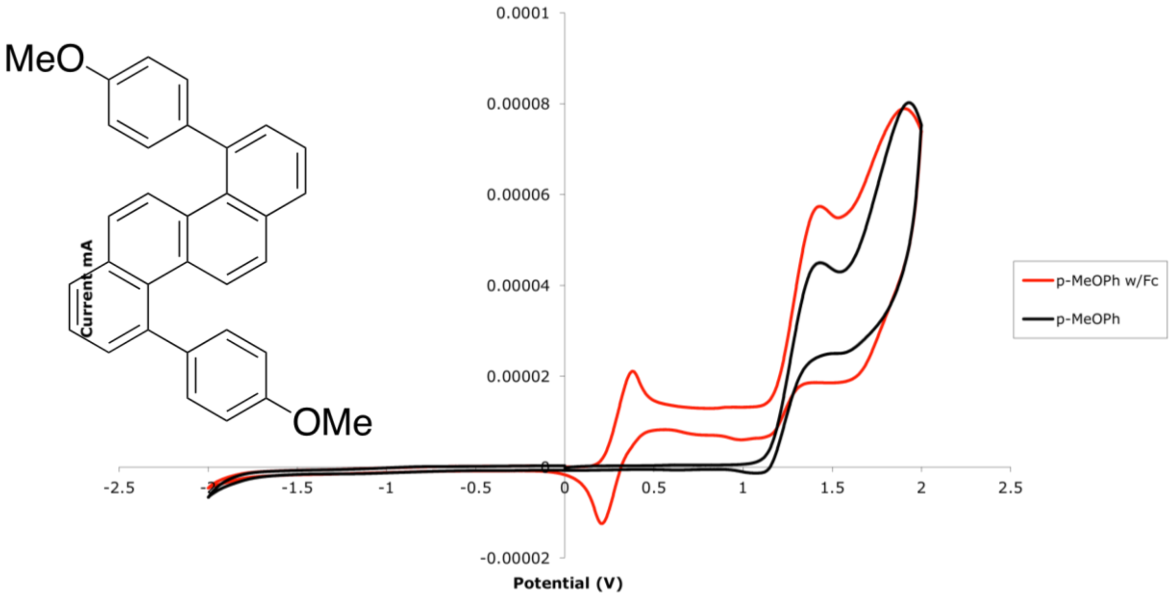

4.5 - 4,10-Bis(3-thienyl)chrysene (19)

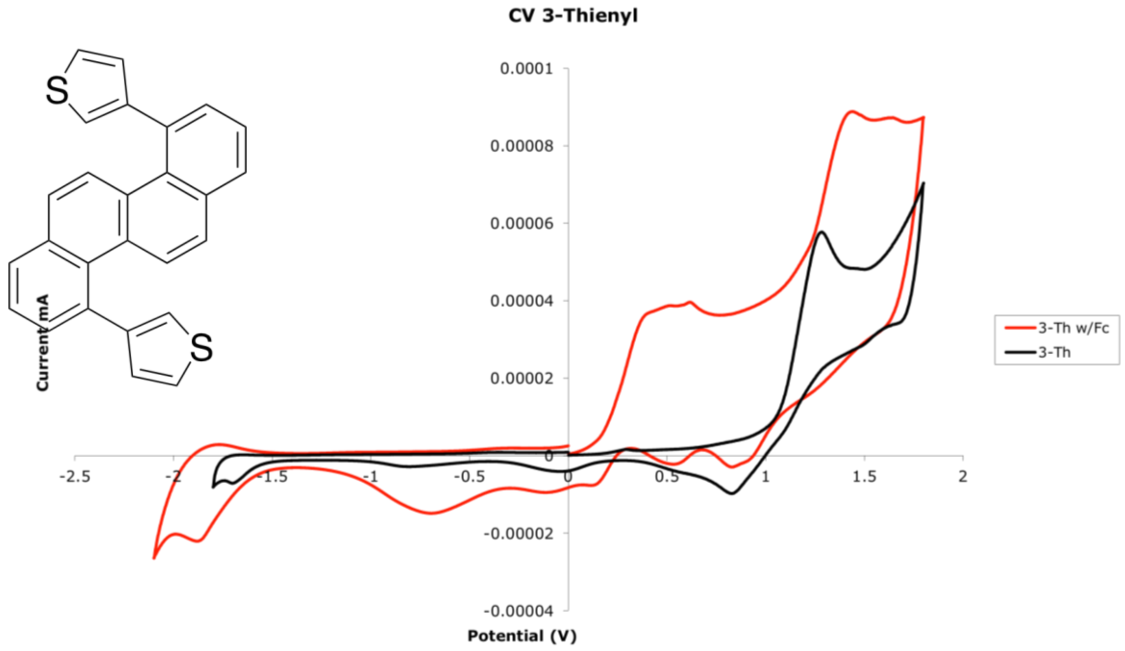

**** 4,10-bis(3-thienyl)chrysene underwent reaction upon voltammetry, leaving a polymeric film on the working electrode.***

4.6 - 4,10-Bis(phenylthio)chrysene (20)

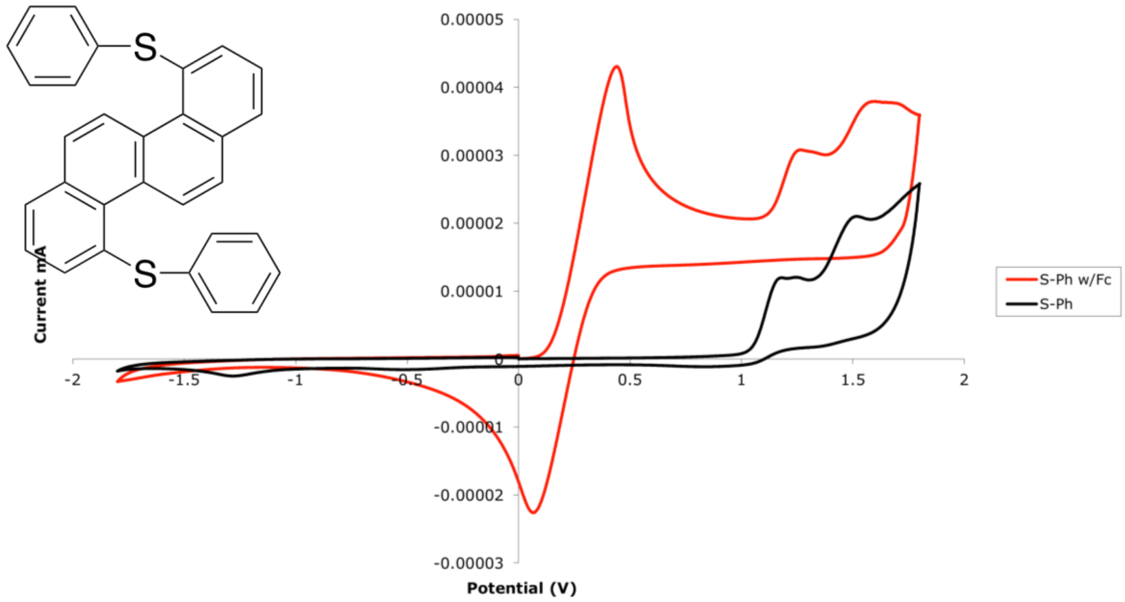

4.7 - 4,10-Bis(2-naphthylthio)chrysene (21)

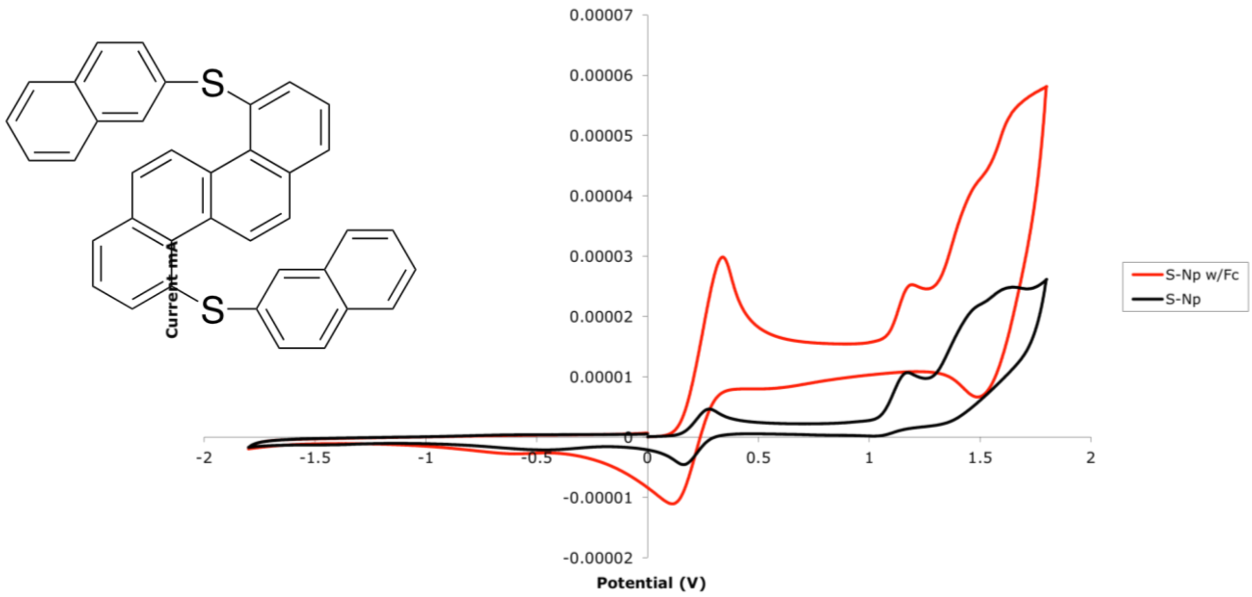

**** Oxidation peak at 0.2 V for naphthyl thiother due to ferrocene.***

4.8 - 4,10-Bis(phenyloxy)chrysene (22)

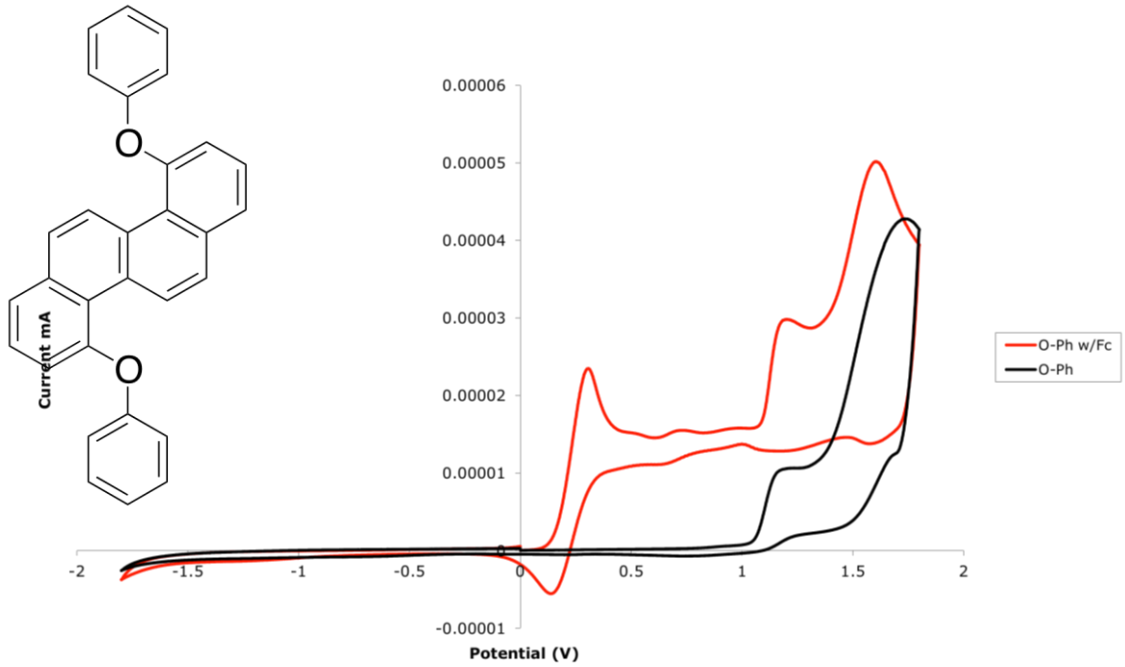

4.9 - 4,10-Bis(oct-1-yn-yl)chrysene (23)

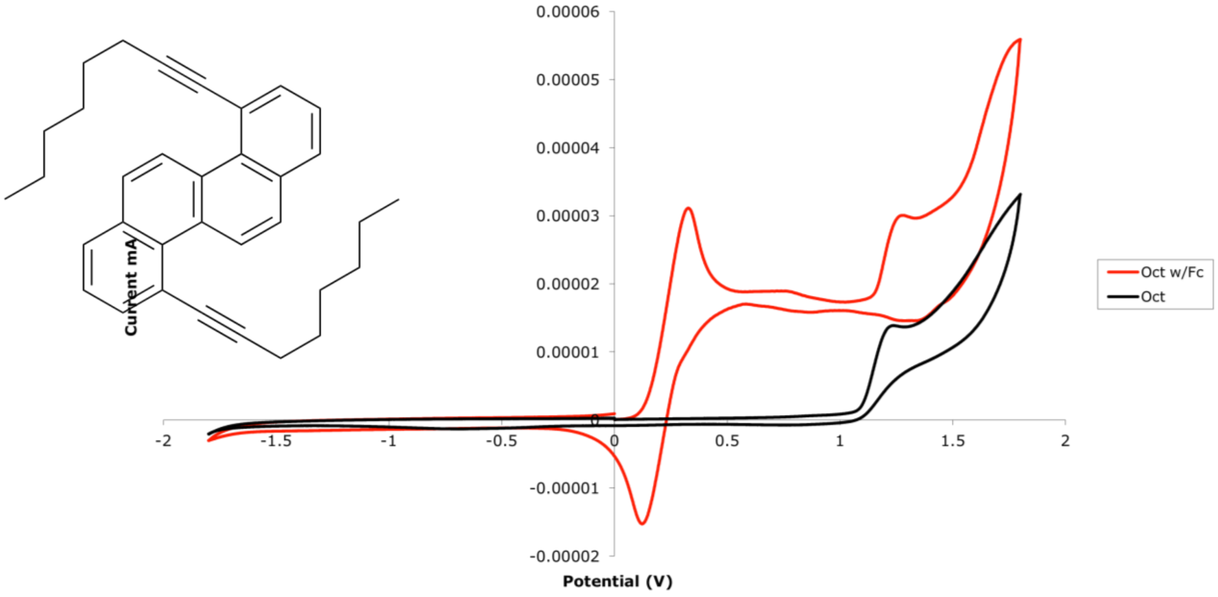

5 - NMR spectra for 4,10-chrysene derivatives

5.0 - 4,10-Dichlorochrysene

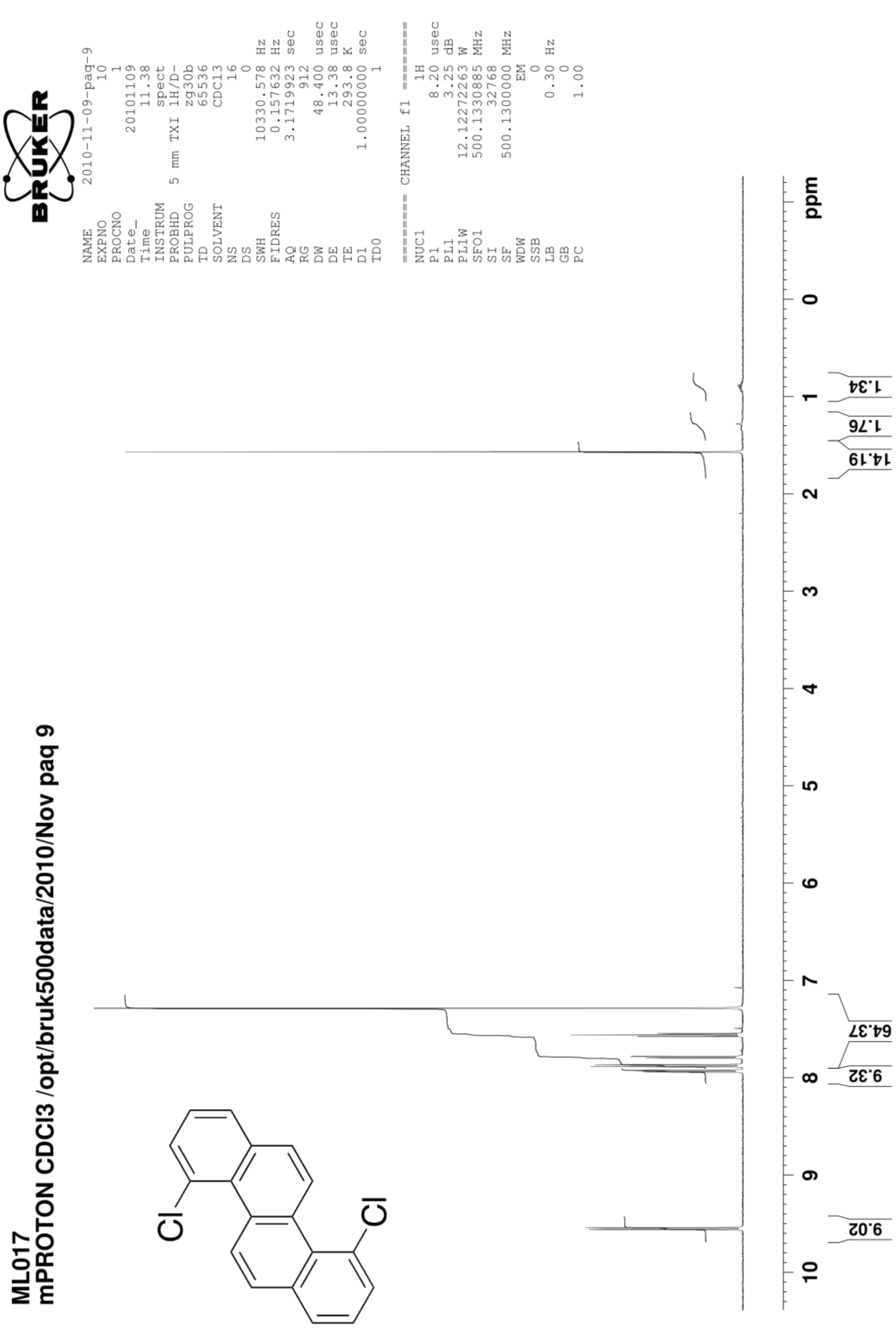

5.1 - 4,10-Dimethylchrysene

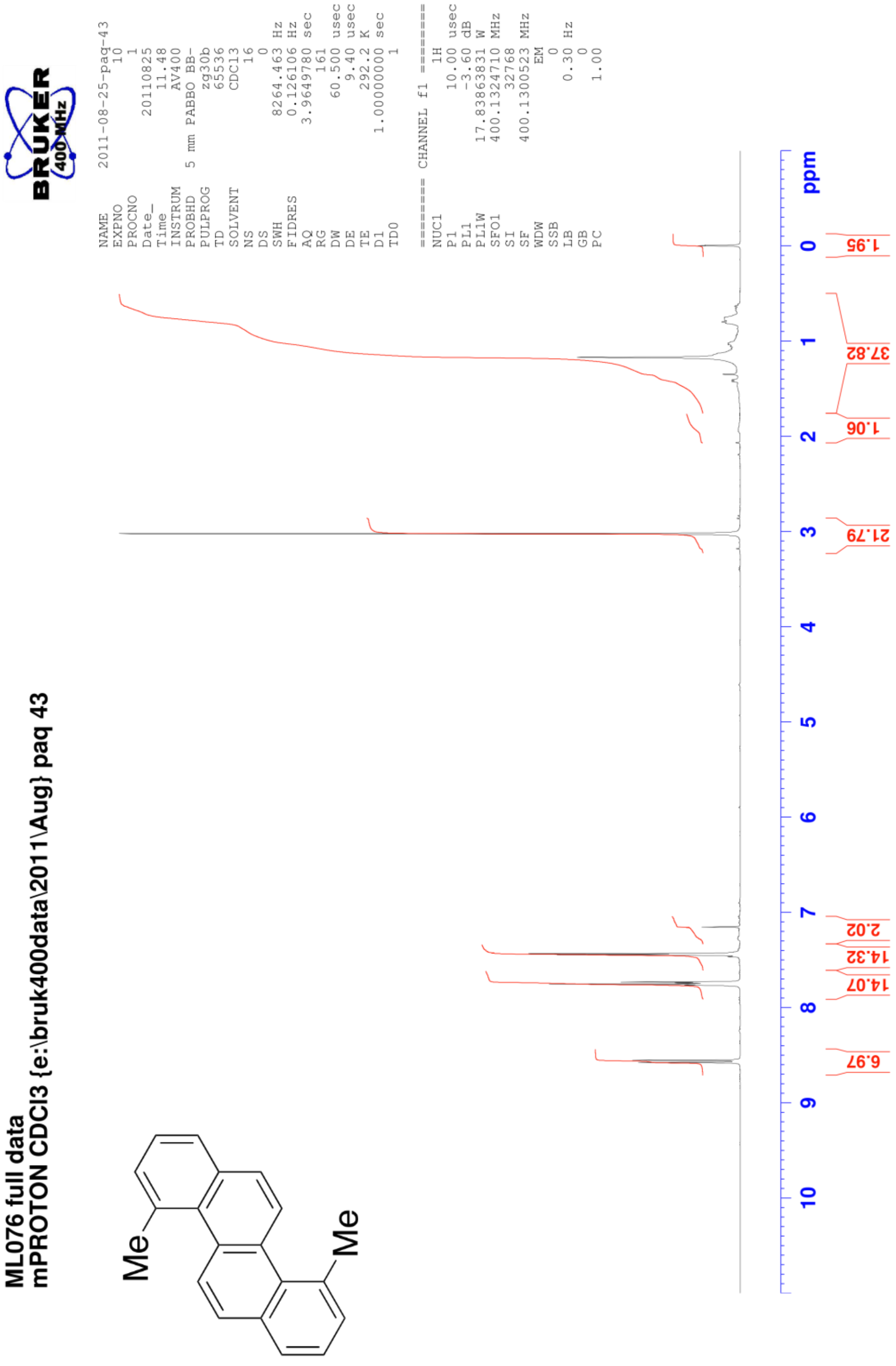

5.2 - 4,10-Diphenylchrysene

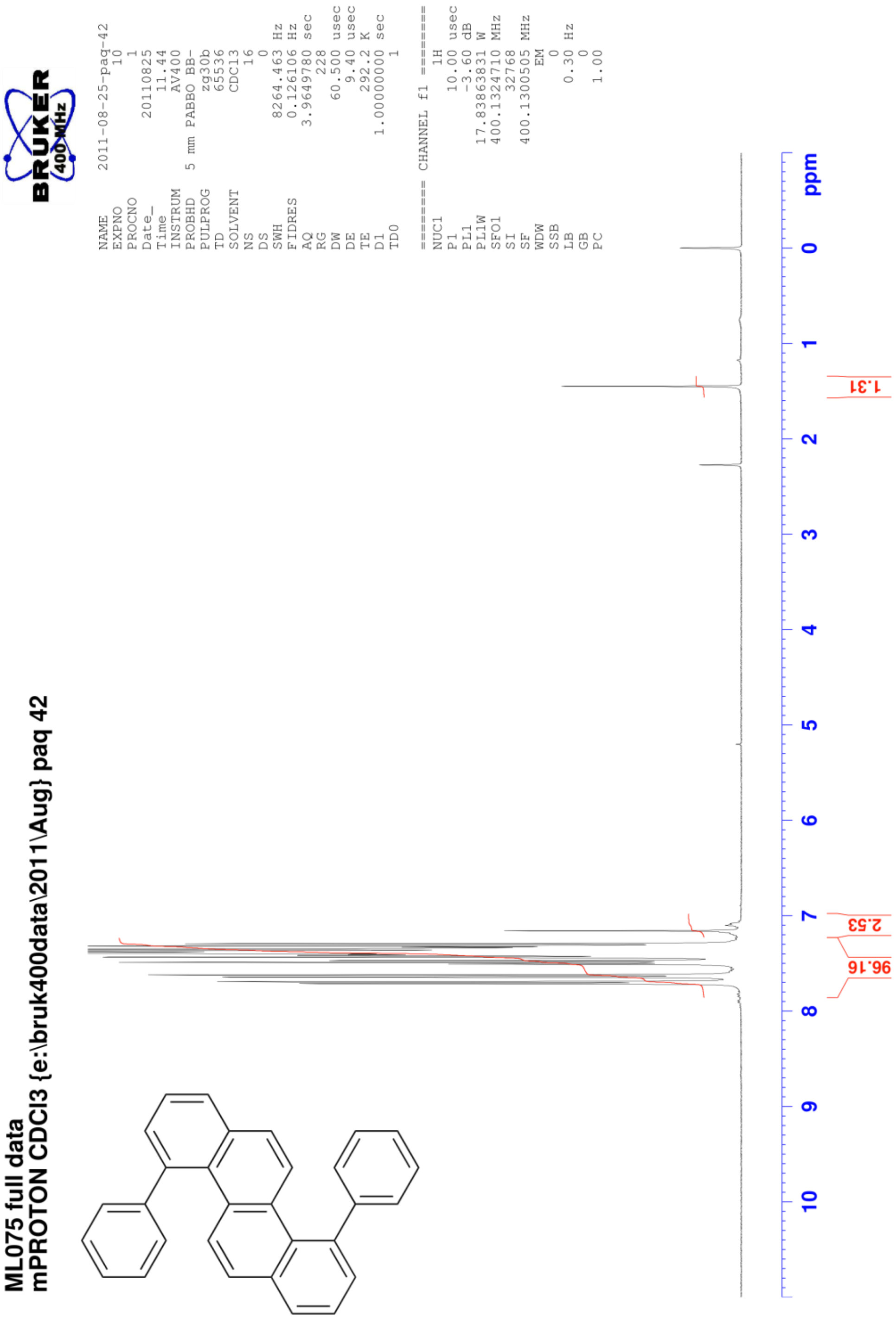

5.3 - 4,10-Bis(4-methoxyphenyl)chrysene

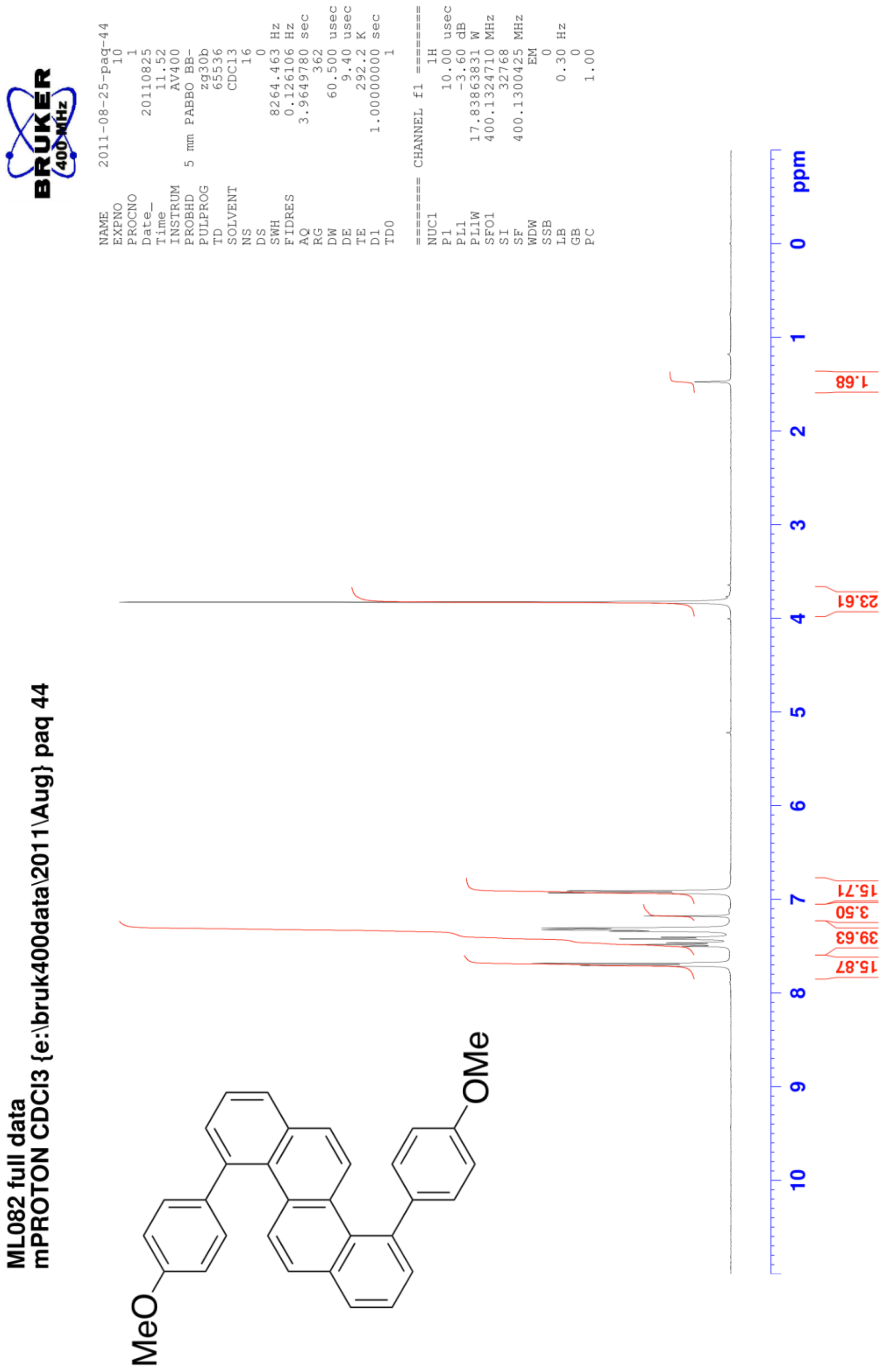

5.4 - 4,10-Bis(1-naphthyl)chrysene

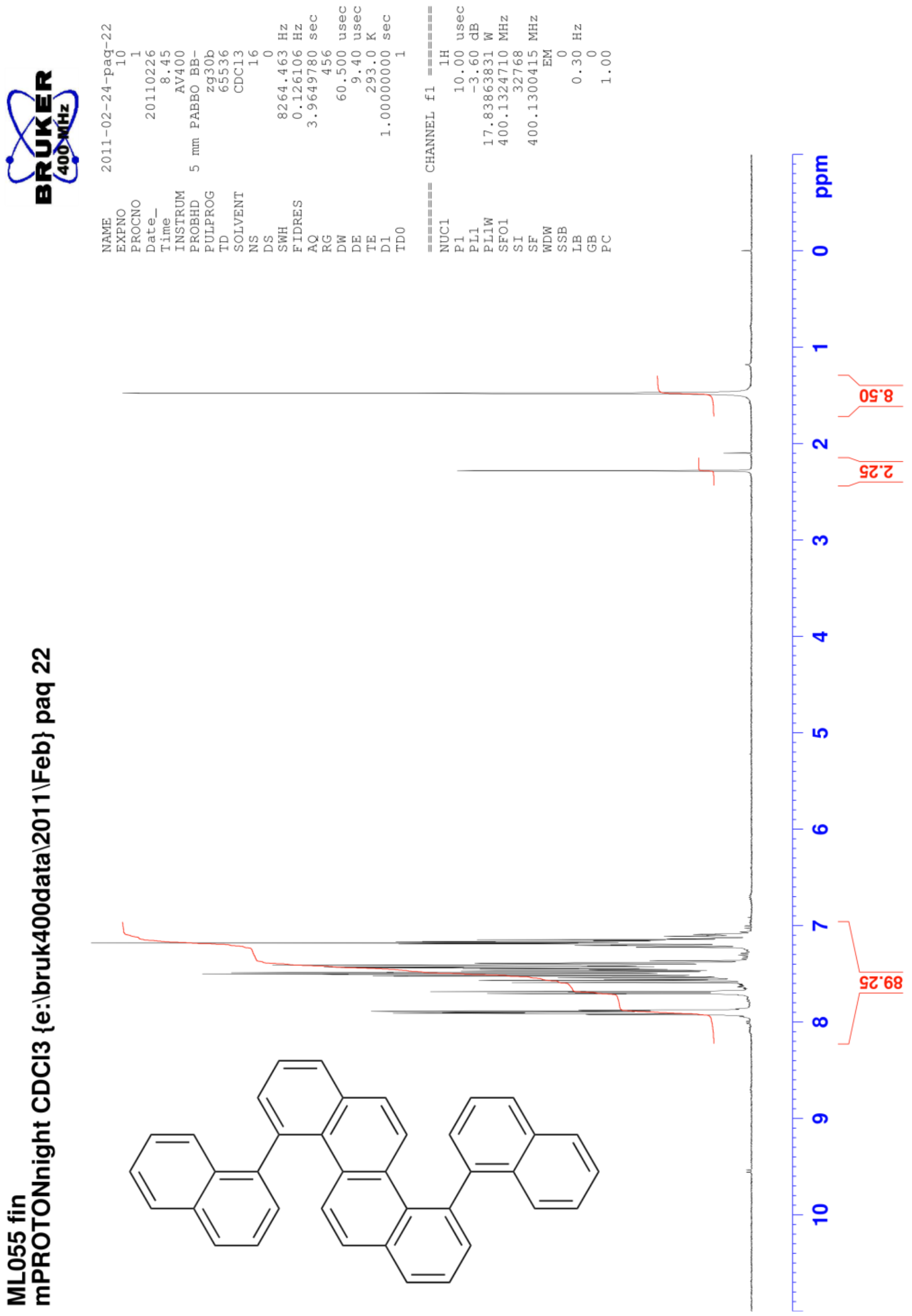

5.5 - 4,10-Bis(3-thienyl)chrysene

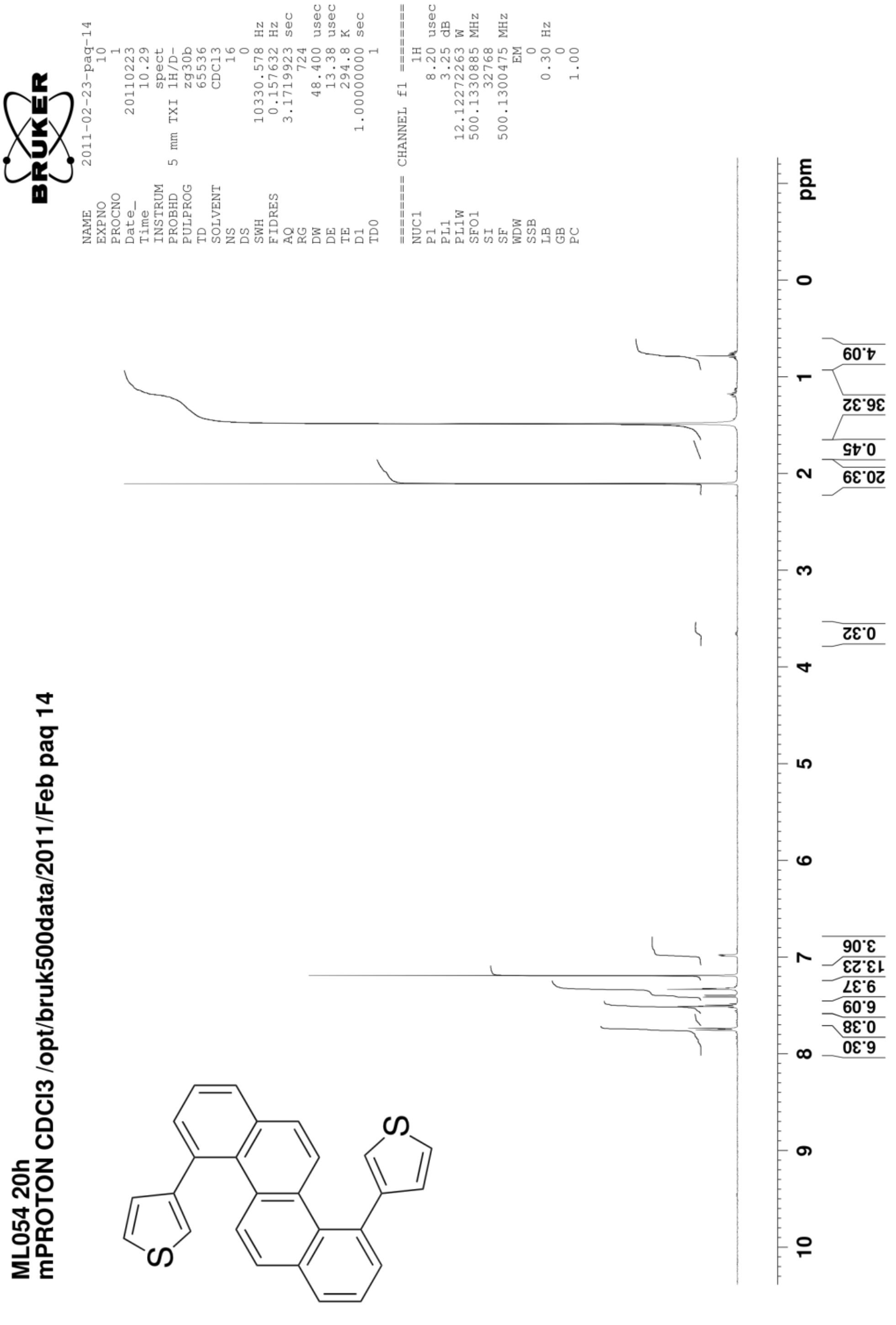

5.6 - 4,10-Bis(phenylthio)chrysene

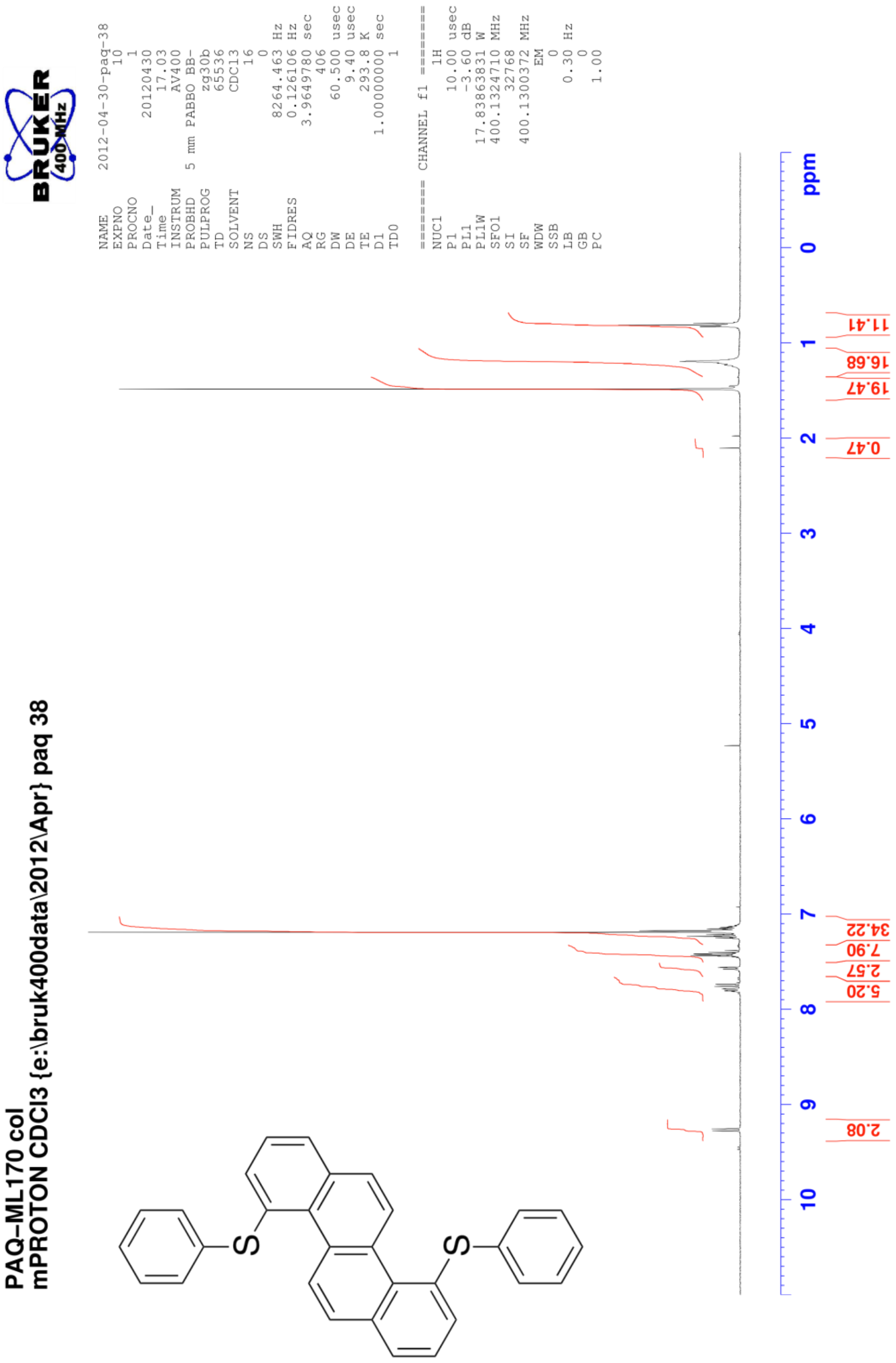

5.7 - 4,10-Bis(2-naphthylthio)chrysene

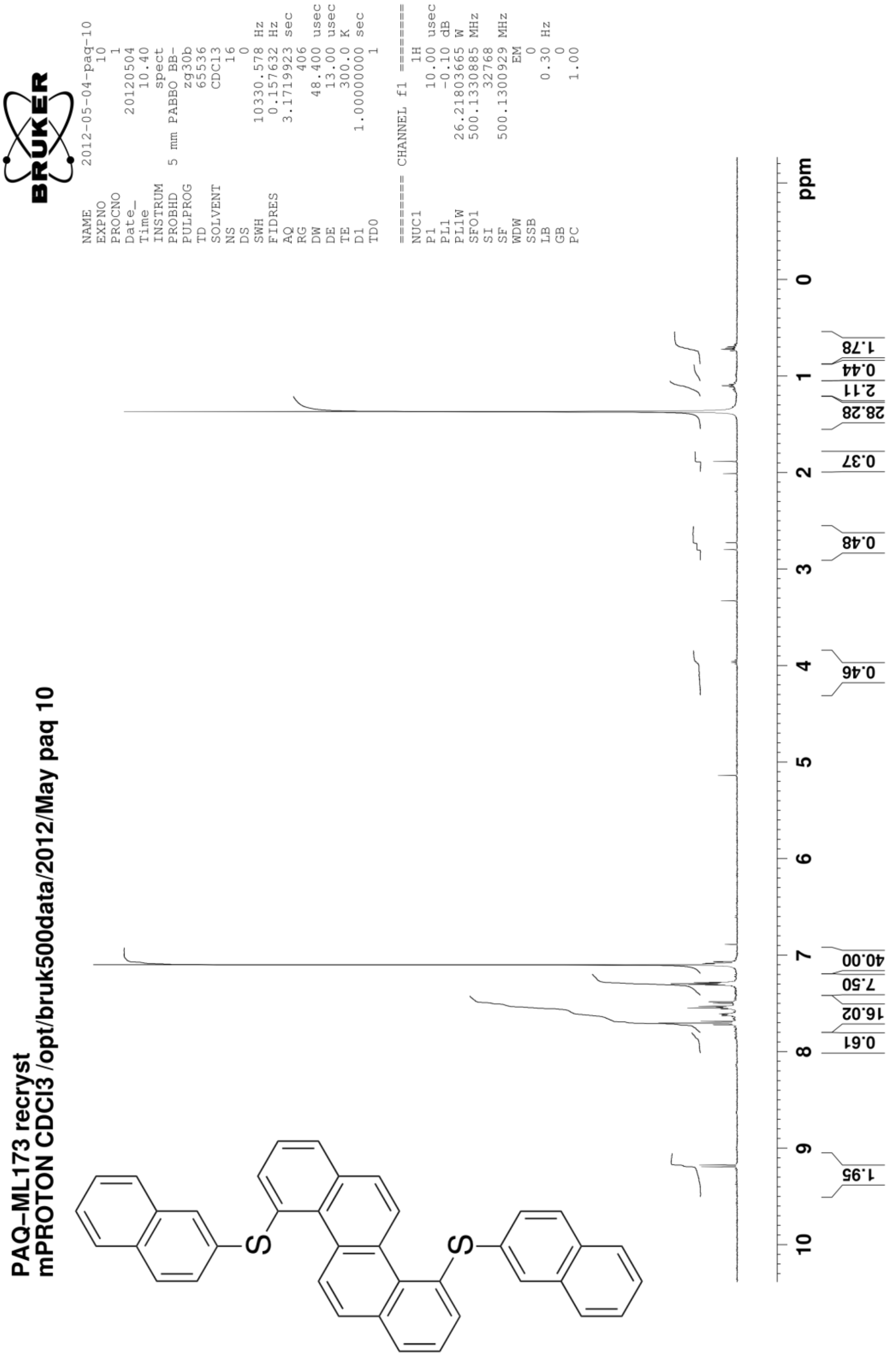

5.8 - 4,10-Bis(phenyloxy)chrysene

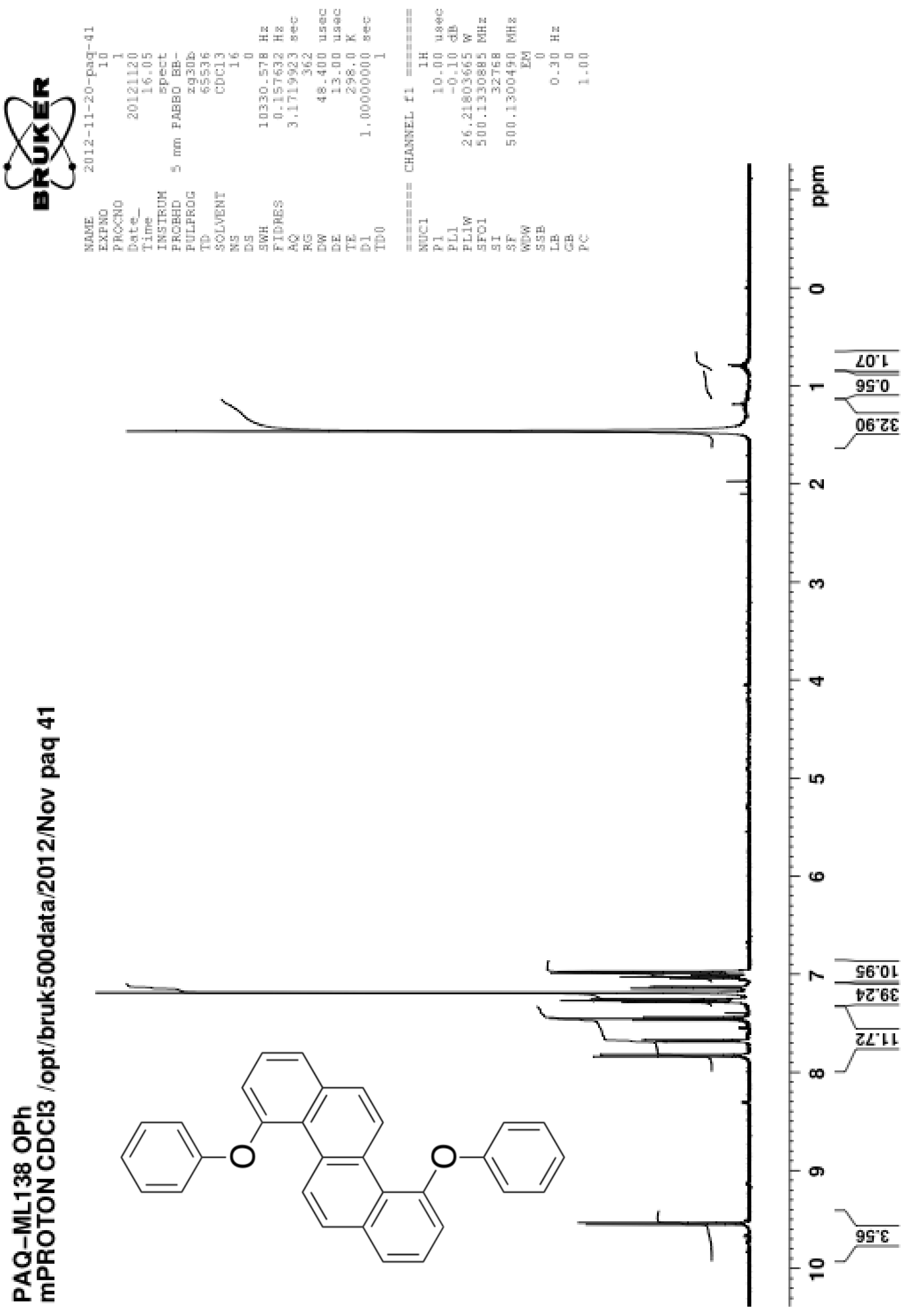

5.9 - 4,10-Bis(Oct-1-yn-1-yl)chrysene

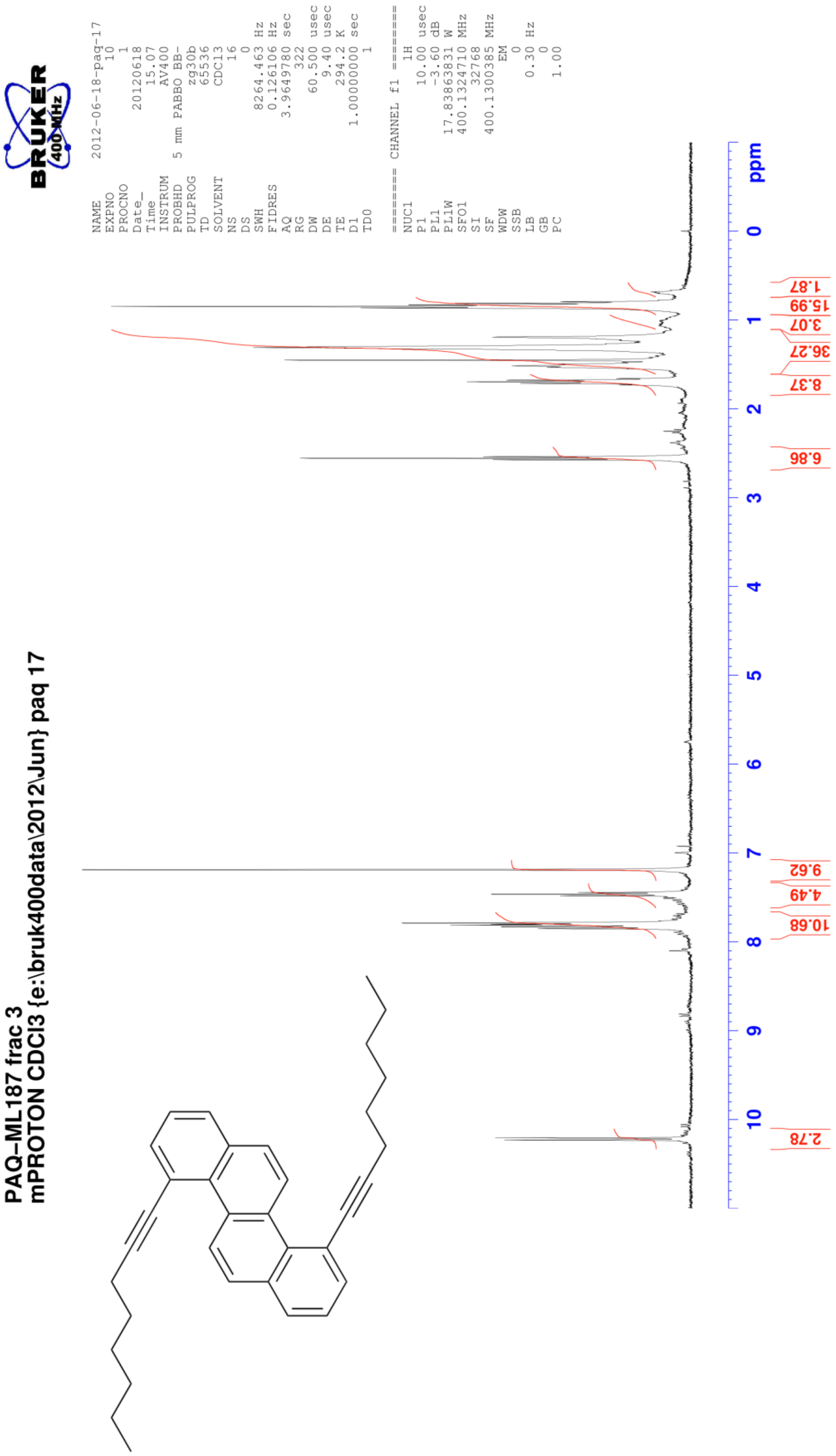

6 - MO Calculations

Each substituted chrysene was fully optimised at the B3LYP/6-31G(d,p) level using the Gaussian09 suite of programs. Using these geometries, the UV/Vis absorption spectra were obtained using time-dependent DFT with the B3LYP functional and the larger 6-311G(d,p) basis set. The MOs of 4,10-diphenylchrysene **14** that correspond to the most intense transition in the calculated electronic spectrum. It can be seen that there is very little contribution from the peripheral phenyl groups in these MOs. This can be attributed to the orthogonal conformation the molecule prefers; it suggests that there is no significant delocalisation from the core to the periphery. In contrast, the MOs associated with the most intense transitions of 4,10-bis(phenylthio)chrysene **18** indicate that there is significant contribution to these MOs from the S-substituent. The appearance of an additional absorption in the UV/vis trace of **18** - a "shoulder" at 310 nm may be indicative of this mode of transition.

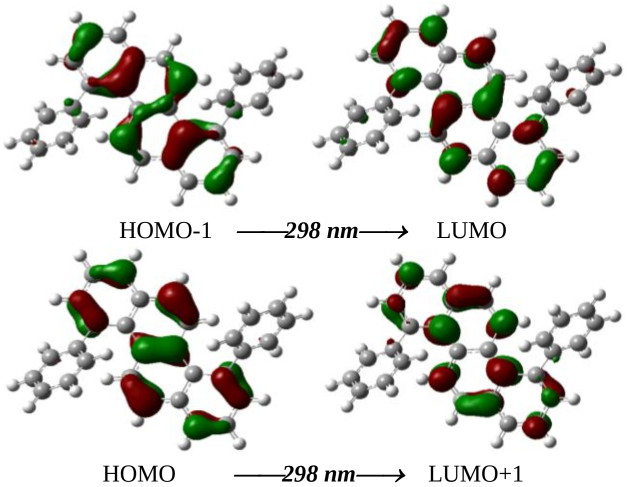

Calculated MOs for the highest intensity transitions of **14**.

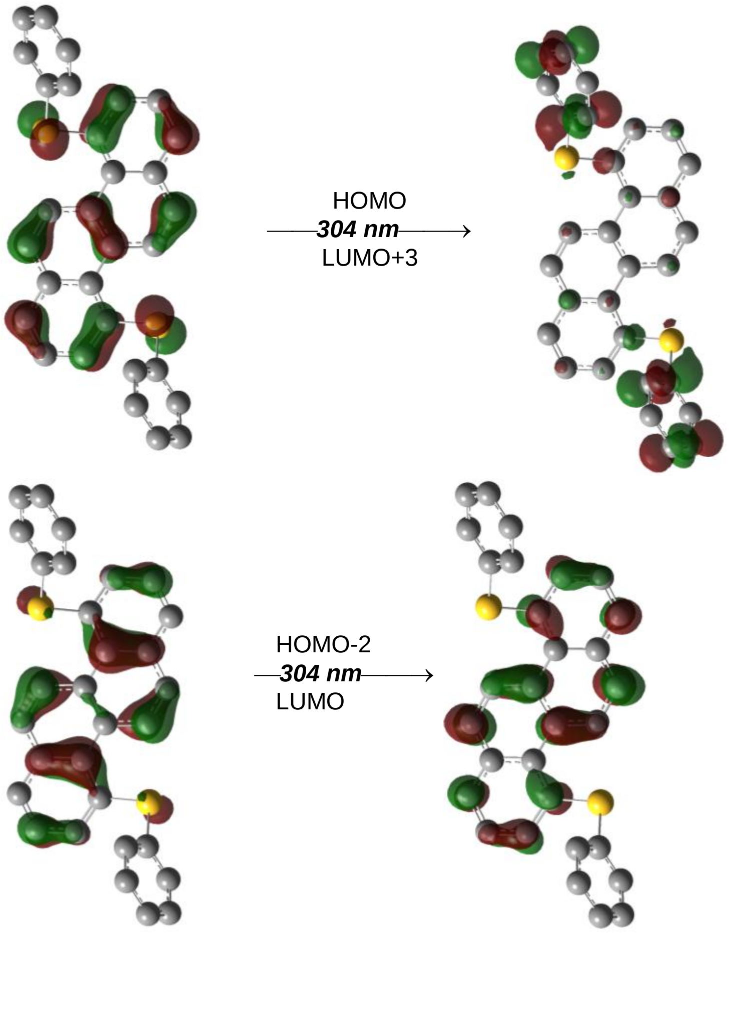

Calculated MOs for the highest intensity transitions of **18**.
